# Multifunctional Electrospun PCL/Starch/n-Al_2_O_3_ Nanocomposites: Potential Antibacterial Wound Dressing Applications

**DOI:** 10.3390/ijms27167117

**Published:** 2026-08-08

**Authors:** Felipe Gutiérrez, Diana Zárate-Triviño, Francisco A. Cataño, Alexander Córdoba, Marcela Saavedra, Esmeralda López, Aline Alfaro, Eliana Rodríguez, Jennifer Leos, Sebastián Zapata, Pedro Orihuela, Paula A. Zapata

**Affiliations:** 1 Grupo Polímeros, Departamento de Ciencias del Ambiente, Facultad de Química y Biología, Universidad de Santiago de Chile, Santiago 9170022, Chile; felipe.gutierrez.t@usach.cl (F.G.); alexander.cordoba@usach.cl (A.C.); marcela.saavedra@usach.cl (M.S.); esmeralda.lopez@usach.cl (E.L.); eliana.rodriguez@usach.cl (E.R.); 2 Laboratory of Immunology and Virology, Faculty of Biological Sciences, Autonomous University of Nuevo Leon, Monterrey 64000, Mexico; diana.zaratetr@uanl.edu.mx (D.Z.-T.); jleosr1343@gmail.com (J.L.); 3 Laboratorio de Inmunología de la Reproducción, Facultad de Química y Biología, Universidad de Santiago de Chile, Santiago 9160000, Chile; facatano@gmail.com (F.A.C.); aline.alfaro@usach.cl (A.A.); 4 Grupo Inteligencia Artificial y Robótica, Departamento de Ingeniería de Sistemas y Computación, Escuela de Ingeniería y Ciencias Básicas, Universidad Escuela de Ingeniería de Antioquia, Envigado 055428, Colombia; pansezapata@gmail.com; 5 Centro para el Desarrollo de la Nanociencia y la Nanotecnología, Santiago 9170022, Chile

**Keywords:** side-by-side electrospun fibers, polycaprolactone, starch, Al_2_O_3_ nanoparticles, wound dressing, antibacterial activity

## Abstract

Multifunctional polymer scaffolds with mechanical support, biocompatibility, and antimicrobial activity are key for next-generation biomedical materials. We report the fabrication of electrospun nanocomposite fibers made from polycaprolactone (PCL), starch, and mesoporous aluminum oxide nanoparticles (n-Al_2_O_3_). Nanoparticles (11 ± 4 nm) were synthesized via a sol-gel method, predominantly comprising γ- and α-Al_2_O_3_ phases. Four fiber systems were fabricated by side-by-side electrospinning: PCL, PCL/starch, PCL/n-Al_2_O_3_, and PCL/starch/n-Al_2_O_3_. SEM analysis confirmed uniform and bead-free fibers in all formulations. Tensile tests showed that the incorporation of starch and nanoparticles improved the mechanical performance compared with neat PCL. In particular, PCL/starch/n-Al_2_O_3_ fibers exhibited increases of 404% in Young’s modulus and 102% in elongation at break. In PBS, starch and n-Al_2_O_3_ enhanced hydrophilicity and accelerated weight loss, with PCL/starch/n-Al_2_O_3_ showing the highest mass loss. Antibacterial tests indicated that only fibers with nanoparticles could inhibit *Staphylococcus aureus* and *Escherichia coli*, with PCL/starch/n-Al_2_O_3_ showing a major effect. Although n-Al_2_O_3_ increased cytotoxicity toward NIH-3T3, starch mitigated this effect, and the ternary scaffold showed no detectable cytotoxicity. Moreover, PCL/starch/n-Al_2_O_3_ exhibited non-hemolytic behavior, enhanced fibroblast migration, and wound-healing-related protein expression. Overall, side-by-side electrospun PCL/starch/n-Al_2_O_3_ scaffold exhibited showed improved mechanical, biological, and antibacterial properties, supporting its potential as a wound-dressing material.

## 1. Introduction

The skin, as the body’s primary protective barrier against the external environment, is continuously exposed to potential damage, making efficient wound healing essential for survival. However, this process can be impaired by factors such as wound size, infection, and patient age [1,2]. Conventional treatments, including skin grafts and the use of amniotic membrane and amniotic fluid, are still widely used but present important limitations: they are expensive, invasive, dependent on donor availability, and may increase the risk of infection, ultimately delaying healing [3].

In this context, there is a growing need for advanced wound dressing materials that not only cover the wound but also actively promote the healing process. Ideally, these materials should maintain a moist environment, allow gas exchange, absorb exudates, and prevent microbial infection [4]. Hydrogel-based materials and polymer-based fibrous scaffolds have been extensively investigated for wound healing applications because they fulfill these requirements while providing a platform for the controlled delivery of therapeutic agents [5,6,7].

Polymer-based fibrous scaffolds typically exhibit high porosity, a large surface-to-volume ratio, and microscale interconnected pores that facilitate nutrient and oxygen transport—features essential for efficient wound dressing materials [8]. Electrospinning has become a highly effective technique for fabricating scaffolds, thanks to its simplicity, cost-efficiency, and versatility in generating polymeric fibers with diameters on the order of nanometers to micrometer [9]. This technique uses a strong electric field to draw fine fibers from a polymer solution or melt. In a typical setup, the polymer solution is placed in a syringe (electrode) and directed toward a conductive collector (counter-electrode). When electrostatic forces overcome surface tension, a charged jet is formed, which stretches and accelerates as the solvent evaporates, resulting in fiber deposition on the collector [10].

Several electrospinning configurations have been developed, including conventional single-needle, multiple-needle, coaxial, sequential multilayer, and side-by-side electrospinning [11,12]. Side-by-side electrospinning allows two formulations to be prepared and delivered independently through adjacent channels and brought into contact during fiber formation, which can facilitate the processing of materials that cannot be readily incorporated into a single homogeneous spinning solution [13]. Compared with coaxial electrospinning, this configuration does not require the establishment and maintenance of a concentric core–shell jet, whose stability depends on the careful optimization of the physicochemical properties and electrospinning conditions of both solutions [11]. Moreover, unlike multiple-needle or sequential multilayer electrospinning, side-by-side electrospinning enables both formulations to be incorporated simultaneously within the same fibers rather than being deposited as separate fiber populations or macroscopic layers [12]. Thus, side-by-side electrospinning provides a relatively simple and versatile strategy for generating multicomponent fibrous scaffolds.

Polycaprolactone is an aliphatic polyester that has been used in the electrospinning technique to obtain scaffolds due to its excellent biocompatibility, low toxicity, and inherent biodegradability [14]. PCL degrades into 6-hydroxycaproic acid, which is safely metabolized by the body [15,16]. However, the intrinsic hydrophobicity of PCL restricts its application in wound dressing materials because adequate hydrophilicity is required to efficiently absorb wound exudate and support cell attachment and proliferation [17]. Insufficient exudate management can result in fluid accumulation at the wound site, impairing cell proliferation, promoting dehydration of the surrounding tissue, and consequently delaying the wound healing process [18]. In addition, the absence of bioactive functional groups on the PCL surface restricts cell adhesion [19], which is essential for cell proliferation and biosynthesis [20]. To overcome these limitations, PCL hydrophilicity and bioactivity can be enhanced through surface modification strategies, including alkaline hydrolysis [21], aminolysis [22], and hydrophilic grafting [23]. However, these approaches present important drawbacks. Surface-modified PCL is prone to surface aging, in which polar groups reorient or diffuse into the polymer bulk, restoring the hydrophobic PCL surface [24]. Likewise, aminolyzed PCL may lose surface amino groups during storage in phosphate-buffered saline or at 37 °C [25], while covalent grafting requires prior surface activation and precise control of functionalization time [26]. An alternative strategy is blending PCL with hydrophilic polymers [27], enabling single-step fabrication (e.g., by electrospinning) while eliminating the need for additional reagents or post-processing steps.

Starch, as the primary energy storage polysaccharide in plants [28], emerges as a promising sustainable alternative for use in conjunction with PCL in the fabrication of electrospun polymer fibers for biomedical applications. This naturally occurring polymer is formed from D-glucose units. It is hydrophilic, biocompatible, non-toxic, non-immunogenic, and biodegradable [29]. PCL–starch electrospun fibers have demonstrated significant improvements in properties relevant to wound care, including increased hydrophilicity, enhanced swelling capacity (supporting exudate management), and improved hemostatic performance, which helps control bleeding [30].

Incorporating metal oxide nanoparticles into electrospun fibers can enhance antimicrobial properties, which are essential for preventing infections in wound dressings. These nanoparticles act through mechanisms such as membrane disruption, ion release, and the generation of reactive oxygen species [31]. Despite the high availability and low cost of aluminum oxide, Al_2_O_3_ nanoparticles (n-Al_2_O_3_) have received relatively little attention with regard to their antimicrobial properties in comparison to other metal oxide nanoparticles [32]. Sikora et al. studied the antimicrobial activity of Al_2_O_3,_ CuO, Fe_3_O_4_, and ZnO nanoparticles against *S. aureus*, *P. aeruginosa*, *C. albicans*, and 4 *E. coli* strains [33]. All nanomaterials exhibited antimicrobial activity with Al_2_O_3_ demonstrated the strongest inhibitory effect against *S. aureus*. In another study, Ansari et al. demonstrated the antibacterial properties of n-Al_2_O_3_ (<50 nm) against strains of *Staphylococcus*: *Staphylococcus aureus* ATCC 25923, methicillin-sensitive *Staphylococcus aureus* (MSSA), and methicillin-resistant *Staphylococcus aureus* (MRSA), the latter known for its resistance to multiple antibiotics [34].

The advantage of using n-Al_2_O_3_ in biomedicine lies in their low toxicity to eukaryotic cells compared to other metal oxide nanoparticles. Zhang et al. investigated the cytotoxic effects of ZnO, TiO_2_, SiO_2_ and Al_2_O_3_ nanoparticles, each with size close to 20 nm, on human fetal lung fibroblasts (HFL1) under in vitro conditions [35]. Across the entire concentration range tested (0.25–1.50 mg/mL), all nanoparticles induced mitochondrial dysfunction, cellular morphological alterations, and cellular apoptosis in a concentration-dependent manner. Among the materials evaluated, ZnO nanoparticles exhibited the highest level of toxicity, followed in decreasing order by TiO_2_, SiO_2_, and Al_2_O_3_, as determined by cell viability assays. Song et al. investigated the oral toxicity of Al_2_O_3_ (nano- and submicron particles with α and γ crystalline phases) and ZnO (nano- and submicron particles) toward Caco-2 intestinal cells and a Caco-2 intestinal epithelium monolayer, evaluating the effects of particle size, dose, crystalline phase, and dissolution [36]. ZnO nanoparticles exhibited substantially higher toxicity than Al_2_O_3_ nanoparticles, inducing significant cytotoxicity at 18 µg/mL, whereas Al_2_O_3_ particles caused only slight toxicity even at 50 µg/mL. Toxicity was primarily governed by dose and particle dissolution, rather than particle size or crystalline phase. ZnO toxicity was mainly attributed to dissolved Zn^2+^ ions, leading to mitochondrial membrane depolarization, apoptosis, and IL-8 production, while Al_2_O_3_ toxicity was associated with ROS generation, G1 cell-cycle arrest, mitochondrial dysfunction, and apoptosis. However, some studies suggest that cytotoxicity depends on the crystalline phase. Nogueira et al. investigated the influence of the crystalline phase on Al_2_O_3_ nanoparticle toxicity in N2A (mouse neuroblastoma) and BEAS-2B (human bronchial epithelial) cells and showed that η-Al_2_O_3_ nanoparticles were more toxic than α-Al_2_O_3_ nanoparticles, likely due to their greater accumulation in cytoplasmic vesicles [37]. Radziun et al. evaluated the cytotoxic effects of γ-Al_2_O_3_ nanoparticles (spherical, 50–80 nm) on mammalian cells, specifically murine fibroblasts and normal human skin fibroblasts, at concentrations of up to 400 µg/mL [38]. Despite the ability of nanoparticles to penetrate the cell membranes of both cell types, they did not significantly induce apoptosis or affect cell viability. Therefore, the incorporation of n-Al_2_O_3_ into PCL/starch electrospun fibers represents a promising strategy for imparting antimicrobial properties, while maintaining the biocompatibility of the resulting material.

Previous studies have demonstrated that n-Al_2_O_3_ enhances the mechanical, antimicrobial, and biological performance of polymer-based composites. Incorporation of n-Al_2_O_3_ (80 nm) increased the tensile strength of polysulfone/polyethylenimine electrospun nanofibers from 0.36 to 0.69 MPa [39]. PLA/Al_2_O_3_ nanoscaffolds exhibited improved flexural, compressive, and modulus properties, with optimal performance observed at 80 ppm n-Al_2_O_3_ [40]. In poly(3-hydroxybutyrate)-chitosan scaffolds, the addition of Al_2_O_3_ nanowires (3 wt%) enhanced tensile strength [41]. In terms of antimicrobial activity, epoxy composites reinforced with n-Al_2_O_3_-embedded banyan fibers showed strong antibacterial effects against *Streptococcus pyogenes*, producing inhibition zones of 12 mm and 9 mm at 100 and 50 µg, respectively, together with significant antibiofilm activity [42]. Likewise, atomic layer deposition of Al_2_O_3_ coatings on poly(methyl methacrylate) improved antifungal performance against *Candida albicans*, with 25–50 nm coatings providing the best balance between efficacy and structural integrity [43]. PLGA/n-Al_2_O_3_ nanocomposites also demonstrated marked antibacterial activity against *Escherichia coli*, achieving growth inhibition above 50% at 0.01% nanoparticle concentration and 74% at 0.1%. Importantly, these nanocomposites showed no detectable cytotoxicity toward SH-SY5Y mammalian cells within the 0.001–0.1% Al_2_O_3_ concentration range [44].

Despite the studies described above, no reports to date have investigated the incorporation of n-Al_2_O_3_ into electrospun PCL/starch fibers or evaluated their antimicrobial activity for potential wound-healing applications. The only closely related study is that of Dong et al. [45], who demonstrated that the incorporation of nanoscale Al_2_O_3_ whiskers into electrospun PCL scaffolds improved the mechanical properties while maintaining biocompatibility. Among the evaluated composites, scaffolds containing 10 wt% Al_2_O_3_ whiskers exhibited the best mechanical performance, achieving a tensile strength of 7.3 MPa and an elastic modulus of 16.1 MPa. Furthermore, in vitro hMSC assays showed comparable cell adhesion, morphology, and proliferation between reinforced and pristine PCL scaffolds, indicating that the addition of Al_2_O_3_ did not adversely affect cellular compatibility.

Based on the studies discussed above, further investigation into the incorporation of n-Al_2_O_3_ nanoparticles into electrospun wound-healing materials is warranted to better elucidate their potential contribution to the physicochemical, mechanical, and biological performance of wound dressings. In parallel, the combination of hydrophobic and hydrophilic polymers, such as polycaprolactone (PCL) and starch, has emerged as a promising strategy for tailoring the properties of electrospun scaffolds intended for skin regeneration. In this context, starch enhances hydrophilicity and biodegradability, whereas PCL provides the mechanical strength and structural stability required to maintain the integrity of the wound dressing throughout the healing process. Building on these considerations, the present study reports the fabrication of electrospun PCL/starch/n-Al_2_O_3_ composite fibers as a novel material for wound-healing applications. To the best of our knowledge, this is the first study to investigate the synergistic integration of a PCL–starch polymer blend with n-Al_2_O_3_ nanoparticles in an electrospun wound dressing. This approach contributes to the design of multifunctional biomaterials by combining the complementary properties of polymer blends and ceramic nanoparticles, supporting their potential use in wound dressing applications. The antimicrobial activity of the scaffolds was evaluated against *Escherichia coli* and *Staphylococcus aureus*. Cytotoxicity was assessed through cell viability assays using NIH-3T3 cell line. The effects of incorporating starch, n-Al_2_O_3_, and their combination into PCL on the structural, morphological and mechanical properties of the fibers were analyzed. Notably, the simultaneous incorporation of starch and n-Al_2_O_3_ enhanced both hardness and ductility. These results demonstrate the potential of PCL/starch/n-Al_2_O_3_ electrospun fibers as biocompatible material with antimicrobial activity and remarkable mechanical properties for biomedical applications, such as wound healing.

## 2. Results and Discussion

### 2.1. Characterization of Al_2_O_3_ Nanoparticles

**Figure 1:**
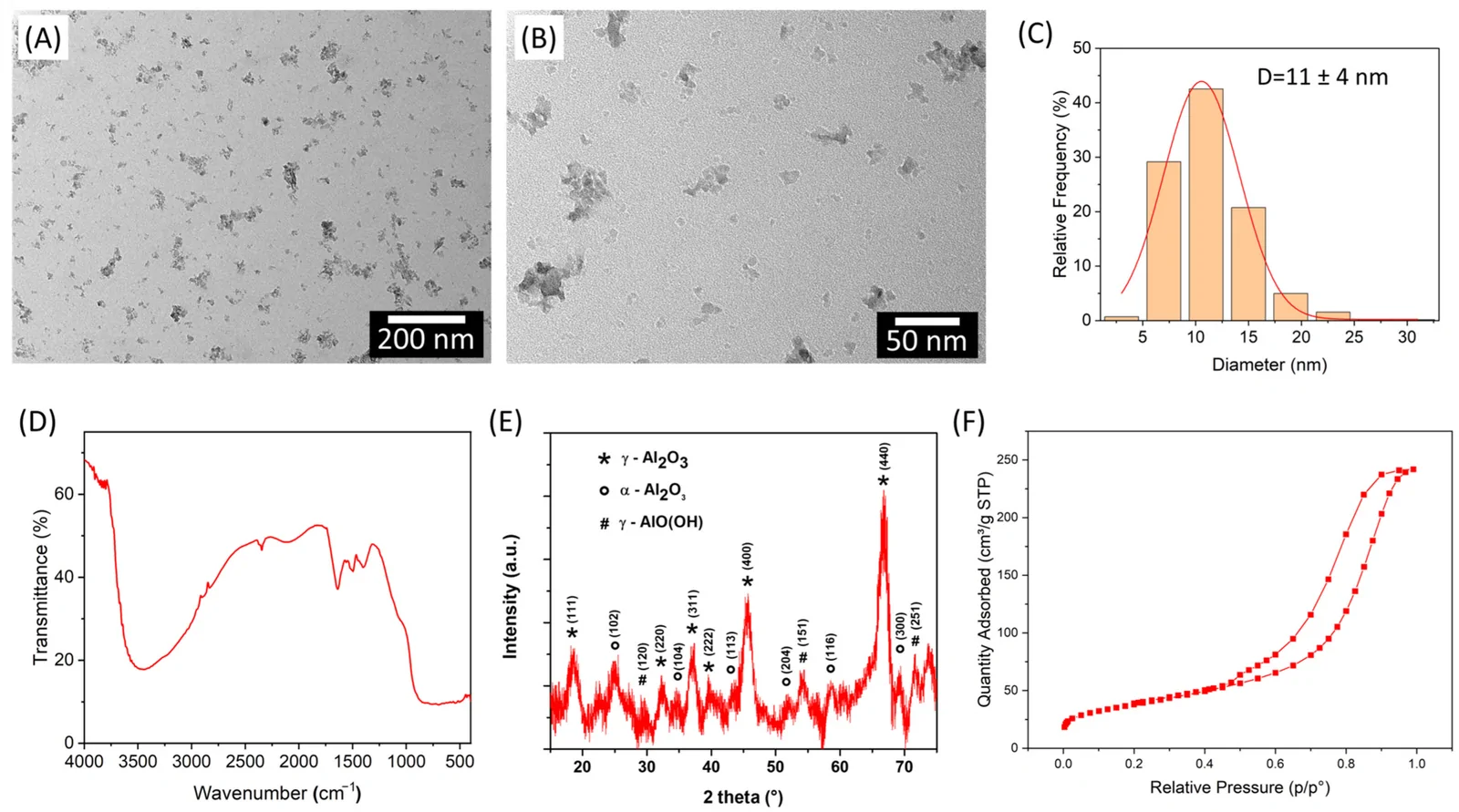
(**A**,**B**) TEM images; (**C**) particle size distribution histogram; (**D**) FT-IR (DRIFTS) spectrum; (**E**) XRD pattern and (**F**) N_2_ isotherm adsorption–desorption of n-Al_2_O_3_.

Figure 1A,B present TEM images of aluminum oxide nanoparticles (n-Al_2_O_3_) synthesized via the sol-gel method. These images confirm the nanometric size of the particles. Figure 1C shows the histogram of particle sizes, in which the average particle size obtained was 11 ± 4 nm.

Figure 1D shows the FT-IR spectrum of n-Al_2_O_3_. The spectrum exhibits a broad band at 3500 cm^−1^, attributed to the stretching vibration of the O-H bond from water molecules adsorbed on Al_2_O_3_. The band located at 1641 cm^−1^ can be attributed to the bending vibration of OH groups. The IR spectrum below 1000 cm^−1^ corresponds to the fingerprint region of Al_2_O_3_. This region is associated with different vibrational modes of the Al_2_O_3_ structure and can be useful in identifying and differentiating between various forms of Al_2_O_3_. Boumaza et al. reported 5 absorption bands in the α-Al_2_O_3_ fingerprint region, located at 386, 457, 484, 608 and 645 cm^−1^ [46]; the band located at 386 cm^−1^ was attributed to the bending of Al octahedrally coordinated by oxygen atoms, while the other bands were attributed to the stretching of Al–O bonds. The research group of Sangor and Al-Ghouti reported three bands below 1000 cm^−1^ for γ-Al_2_O_3_; 910, 804 and 577 cm^−1^ [47], which were associated with asymmetric stretching, symmetric stretching and bending vibrations, respectively. The IR spectrum of the n-Al_2_O_3_ below 1000 cm^−1^ is characterized by a broad and intense band. The width of the band may be due to the coexistence of various crystalline phases within the sample; that is, the spectrum in this region reflects the overlap of the IR spectra of different polymorphs of Al_2_O_3_.

Figure 1E shows the X-ray diffraction patterns of the n-Al_2_O_3_ powder. Diffraction peaks located at 18.6°, 32.3°, 37.2°, 39.6°, 45.7°, and 66.8°, are associated with the crystal planes (111), (220), (311), (222), (400) and (440) of the cubic γ-Al_2_O_3_ phase, respectively. Moreover, additional diffraction peaks characteristic of the hexagonal α-Al_2_O_3_ phase are located at 25.0°, 34.7°, 43.5°, 52.0°, 58.4°, and 69.0°, corresponding to the (102), (104), (113), (204), (116) and (300) crystal planes, respectively [48]. The remaining diffraction peaks located at 29.0°, 54.2° and 71.7° may possibly be attributed to the (120), (151) and (251) crystal planes of the orthorhombic γ-AlO(OH) phase [49]. Boehmite, γ-AlO(OH), is a product of the sol-gel synthesis from aluminum alcoholates, which is subsequently calcined to produce various alumina phases. The obtained phases depend on factors such as formation temperature, particle size and moisture content [50]. X-ray diffraction analysis indicated that, from the synthesis procedure, mainly γ-Al_2_O_3_ and α-Al_2_O_3_ were obtained as products, as well as possibly remaining boehmite. The average crystallite size was estimated using the Scherrer equation (Equation (1)), selecting only well-resolved diffraction peaks, without overlapping and with clear baseline to ensure reliable estimates. For the γ-Al_2_O_3_ phase, the average crystallite sizes measured along the (400) and (311) directions were 5.65 nm and 5.89 nm, respectively, confirming the nanocrystalline nature of the material.

The N_2_ isotherm adsorption is shown on Figure 1F. For n-Al_2_O_3_, the surface area (SSA) calculated with the BET method was 135.5 ± 0.5 m^2^ g^−1^, with a pore volume of 0.378 cm^3^ g^−1^, while the average diameter pore was 10.82 nm, indicating a mesoporous structure (International Union of Pure and Applied Chemistry—IUPAC). The obtained SSA value is low compared to pure γ-Al_2_O_3_, whose surface area can reach up values close to 300 m^2^ g^−1^ [51]. This result is mainly due to a phase mixture between γ-Al_2_O_3_ and α-Al_2_O_3_, where α-Al_2_O_3_ is the phase with the lowest SSA (2–20 m^2^ g^−1^) [51].

Following the same synthesis procedure at a lower calcination temperature (400 °C), Grant et al. reported a surface area, pore width, and pore volume of 338 m^2^ g^−1^, 6.9 nm, and 0.39 cm^3^ g^−1^, respectively [52]. The larger pore size and corresponding decrease in surface area observed in our work could be attributed to the faster thermal decomposition of the Pluronic F-127 template at higher calcination temperatures [53], as well as from the structural shrinkage of the material that becomes more pronounced under these conditions.

### 2.2. Electrospun Fibers Characterization

#### 2.2.1. Morphological Characterization

**Figure 2:**
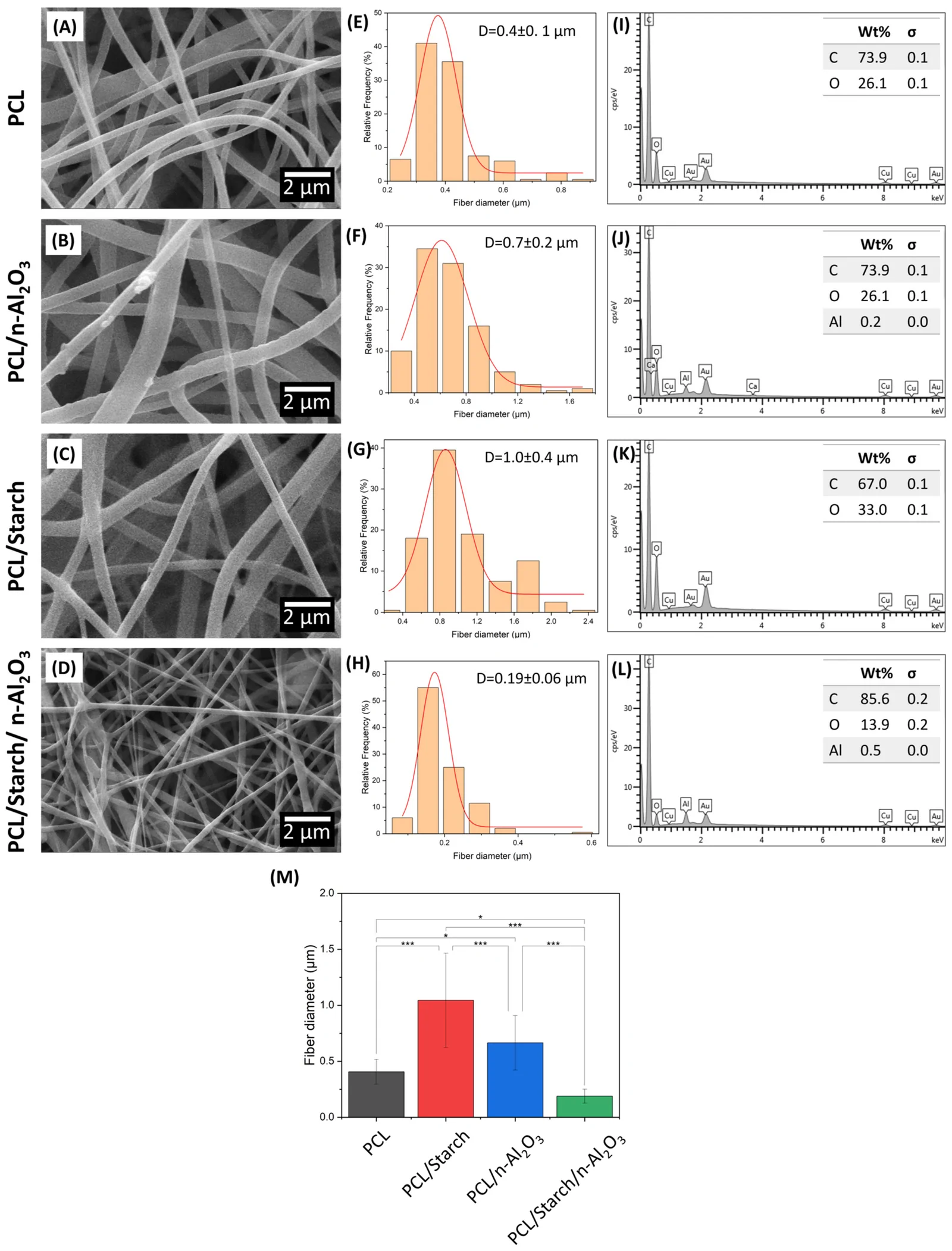
(**A**–**D**) SEM micrographs, (**E**–**H**) fiber diameter distributions, and (**I**–**L**) EDS spectra of electrospun nanofibrous scaffolds: (**A**,**E**,**I**) PCL, (**B**,**F**,**J**) PCL/n-Al_2_O_3_, (**C**,**G**,**K**) PCL/starch, (**D**,**H**,**L**) PCL/starch/n-Al_2_O_3_. (**M**) Statistical differences were assessed by one-way ANOVA followed by Bonferroni’s multiple-comparison test. Data are presented as mean ± SD; *p* < 0.05 (*), *p* < 0.001 (***).

**Figure 3:**
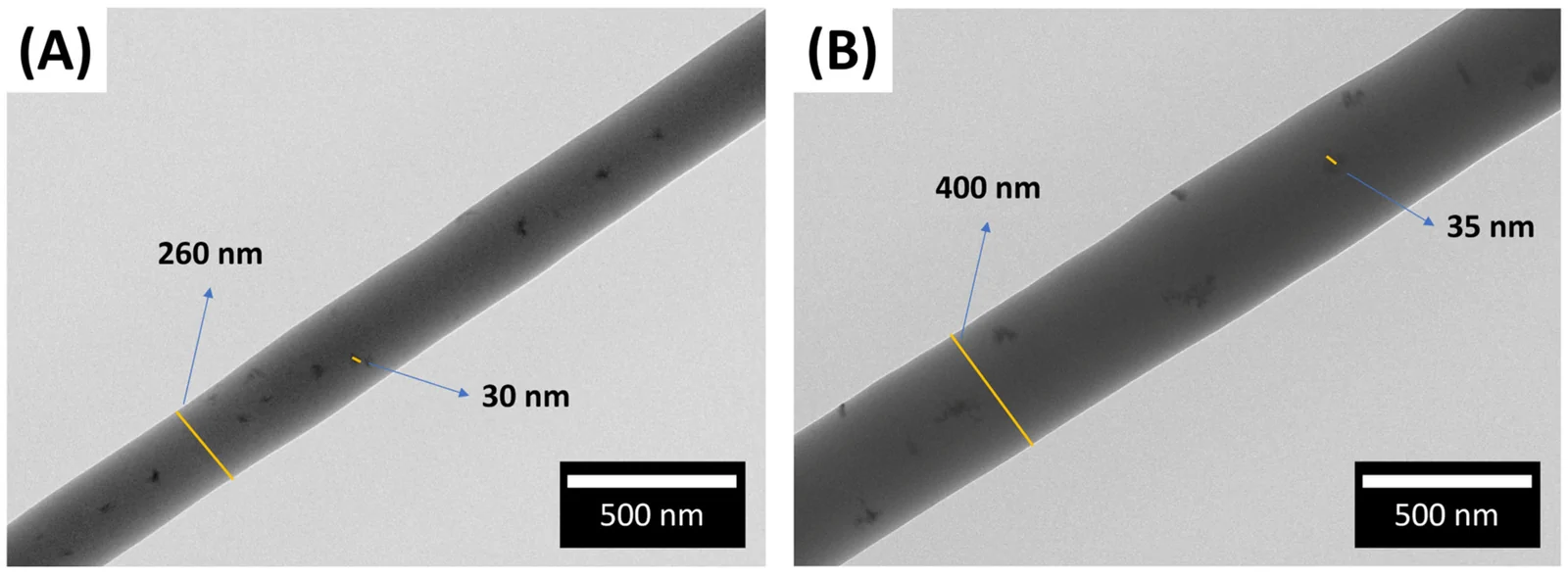
TEM micrographs of electrospun (**A**) PCL/n-Al_2_O_3_ and (**B**) PCL/starch/n-Al_2_O_3_ fibers collected directly onto TEM grids.

To study the morphology and structure of the electrospun fibers, the samples were analyzed by SEM. Figure 2A–D shows the SEM micrographs and respective diameter histograms of electrospun PCL, PCL/n-Al_2_O_3_, PCL/starch and PCL/starch/n-Al_2_O_3_ fibers. SEM images showed that polymeric fibers were obtained satisfactorily in all the polymer composition used. The fibers are characterized by a smooth surface free of beads, except for PCL/n-Al_2_O_3_ fibers in which some agglomerates of Al_2_O_3_ nanoparticles are observed on the surface. These agglomerates are not observed in the PCL/starch/n-Al_2_O_3_ fibers; indicating that, in this case, there was a greater dispersion of the nanoparticles during the electrospinning process. This observation is consistent with our previous work [54], where we found that calcium oxide nanoparticles (n-CaO) were more uniformly dispersed in electrospun PCL/starch/n-CaO fibers than in PCL/n-CaO fibers. This behavior was attributed to the presence of hydroxyl groups in starch; consequently, n-CaO interacts more strongly with starch than PCL. The hydroxyl groups in starch can form hydrogen bonds with metal oxide nanoparticles, acting as a stabilizing agent that prevents nanoparticle agglomeration [55]. Elemental analysis of the fibers using energy dispersive spectroscopy (EDS) confirmed the presence of aluminum in the PCL/n-Al_2_O_3_ and PCL/starch/n-Al_2_O_3_ samples. Furthermore, TEM micrographs (Figure 3) showed that n-Al_2_O_3_ was distributed homogeneously in PCL/n-Al_2_O_3_ and PCL/starch/n-Al_2_O_3_.

The average fiber diameters obtained were 0.4 ± 0.1, 0.7 ± 0.2, 1.0 ± 0.4 and 0.19 ± 0.06 µm for PCL, PCL/n-Al_2_O_3_, PCL/starch and PCL/starch/n-Al_2_O_3_, respectively. In Figure 2M, the statistical comparison of fiber diameters shows that the composition of the solutions fed to the output needles has a significant effect on the resulting fiber dimensions. The first effect observed is that the addition of starch to PCL increases the diameter of the fibers. This same effect was reported by Komur et al. In this study, the researchers prepared PCL/starch fibers by electrospinning using a co-axial configuration and found that increasing the starch content leads to enlarged fiber diameters, which was attributed to a higher solution viscosity [56]. High viscosities of the precursor solution prevent the stretching of the polymer solution jet under the action of the electric field. The smaller the stretching, the larger the diameter of the fibers [56]. Indeed, the molecular weight of potato starch is in the range of 10^6^–10^7^ g/mol [57], which is orders of magnitude higher than the molecular weight of PCL used in this study (8 × 10^4^ g/mol). Therefore, the viscosity of the jet increases when starch is incorporated.

Regarding the influence of n-Al_2_O_3_ on fiber diameter, nanoparticles alter the diameter of the fibers through their impact on solution viscosity and conductivity. First, the addition of n-Al_2_O_3_ to PCL increases the fiber diameter; again, this increase could be explained by the increase in solution viscosity caused by the presence of nanoparticles. However, an opposite effect was observed when nanoparticles were incorporated into PCL/starch fibers; specifically, the addition of nanoparticles led to a reduction in fiber diameter. It has been reported that the incorporation of Al_2_O_3_ nanoparticles decreases the diameter of electrospun polyacrylonitrile fibers [58]. This decrease could be attributed to increase in the electrical conductivity of the solution due to nanoparticle dispersion. A solution with higher conductivity favors a higher charge density in the jet, which in turn enhances the stretching and thinning of the jet during electrospinning, resulting in thinner fibers. The increase in conductivity arises from the formation of an electric double layer around nanoparticles [59], which is more pronounced in ionizable, high-dielectric solvents (AF, ε_r_ = 58.5) than in non-ionizable, low-dielectric solvents (DCM, ε_r_ = 8.93) [60]. Nanoparticles modify fiber diameter by increasing both viscosity and conductivity; however, increases in electrical conductivity have been observed to have a more significant influence on diameter than increases in viscosity [58].

#### 2.2.2. Infrared Spectroscopy Characterization

**Figure 4:**
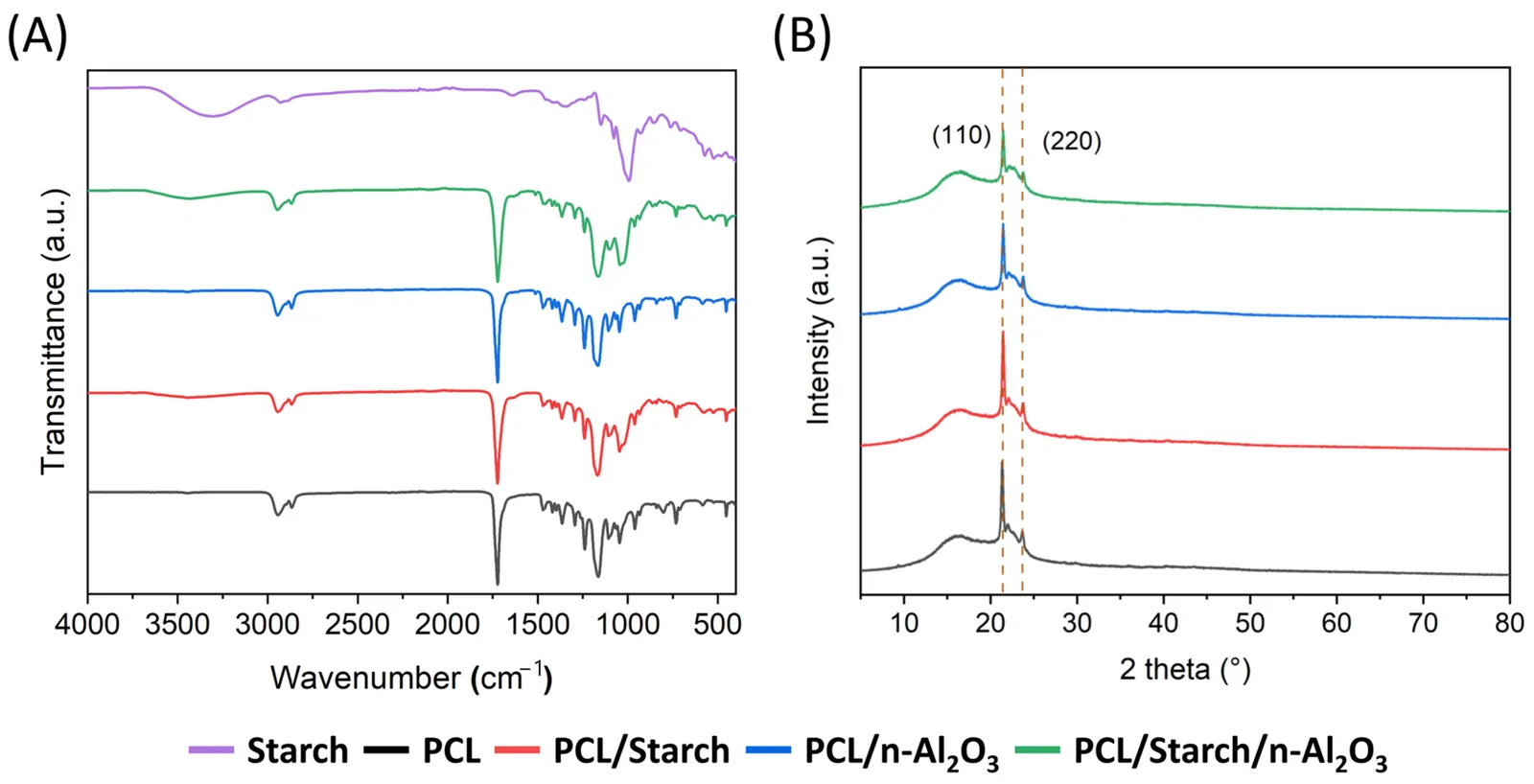
FT-IR (**A**) and XRD (**B**) spectra of electrospun PCL/starch/n-Al_2_O_3_, PCL/n-Al_2_O_3_, PCL/starch, and PCL fibers. For comparison, the FT-IR spectrum of starch powder is also included.

To identify the presence of functional groups in the materials based on electrospun fibers, IR spectra were taken; the results are shown in Figure 4. Since all the materials obtained are mainly composed of PCL, the spectra show similar characteristics defined by the functional groups present in the PCL. First, bands were located at 2944 cm^−1^ and 2861 cm^−1^, which are related to the stretching bands of CH_2_ groups. Another band at 1723 cm^−1^ is attributed to the C=O stretching vibrations of the ester carbonyl group. The bands at 1240 cm^−1^ and 1166 cm^−1^ are associated with C-O-C symmetric and asymmetric stretching, while the band at 1294 cm^−1^ is related to C-O and C-C stretching in crystalline phase [61].

Regarding the electrospun fibers containing PCL and starch, no significant new bands were observed in the IR spectra that could be associated with starch. This may be due to the overlapping of the bands. Only PCL/starch/n-Al_2_O_3_ shows a wide band centered around 3400 cm^−1^, which corresponds to the stretching vibration of the hydroxyl groups; that is, the O-H stretching band is more significant when n-Al_2_O_3_ is incorporated. It is well known that the formation of hydrogen bonds leads to an increase in the intensity of the O-H stretching bands in the IR spectra [62], thus indicating the formation of hydrogen bonds between the OH groups of starch and n-Al_2_O_3_.

It is noteworthy that the dissolution of starch in FA can trigger two reactions: the esterification to form starch formate and the hydrolysis of the glycosidic bonds. In pure FA, these reactions are strongly temperature dependent; below 20 °C the degrees of substitution are low (1.5–1.6) and the destructuration by hydrolysis is not significant [63]. Giri Dev and Hemamalini obtained PCL, PCL/starch (1:1) and PCL/starch formate (1:1) electrospun fibers [30]. In this report, the IR spectra of the PCL and PCL/starch presented similar characteristics, indicating that in the PCL/starch blends, the IR spectrum is mostly determined by PCL. Our results are consistent since all IR spectra were similar to those of PCL fibers. Regarding the PCL/starch formate, Giri Dev and Hemamalini observed a new band at 1712 cm^−1^, which was attributed to starch esterification. Our results showed that for PCL/starch and PCL/starch/n-Al_2_O_3_ materials, no bands attributable to starch esterification were observed, possibly due to low starch content (ca. 25 wt%).

Figure 4B shows the X-ray diffraction spectra of the different electrospun polymer fibers. All spectra display diffraction peaks corresponding to the (110) and (220) planes of the orthorhombic phase of PCL, observed at approximately 21.4° and 23.8°, respectively [64]. The diffraction patterns of PCL/starch and PCL/starch/n-Al_2_O_3_ fibers exhibit no additional peaks compared to those of pure PCL fibers, indicating that the starch in the fibers is in an amorphous structure. Furthermore, the absence of diffraction peaks associated with Al_2_O_3_ can be attributed to the low n-Al_2_O_3_ content in the PCL/n-Al_2_O_3_ and PCL/starch/n-Al_2_O_3_ fibers.

The crystal sizes were obtained from the Scherrer equation (Equation (1)) using the (110) peak. Results are shown in Table 1. As can be concluded, PCL, PCL/starch, and PCL/n-Al_2_O_3_ fibers have crystallite sizes close to 34 nm, with no differences between them. In contrast, PCL/starch/n-Al_2_O_3_ fibers have a slightly smaller crystal size of 32.28 nm. This smaller particle size can be attributed to the confinement effect caused by the smaller diameter of PCL/starch/n-Al_2_O_3_ fibers which can restrict the mobility of polymer chains required for crystallization [65].

#### 2.2.3. Thermal Characterization

**Figure 5:**
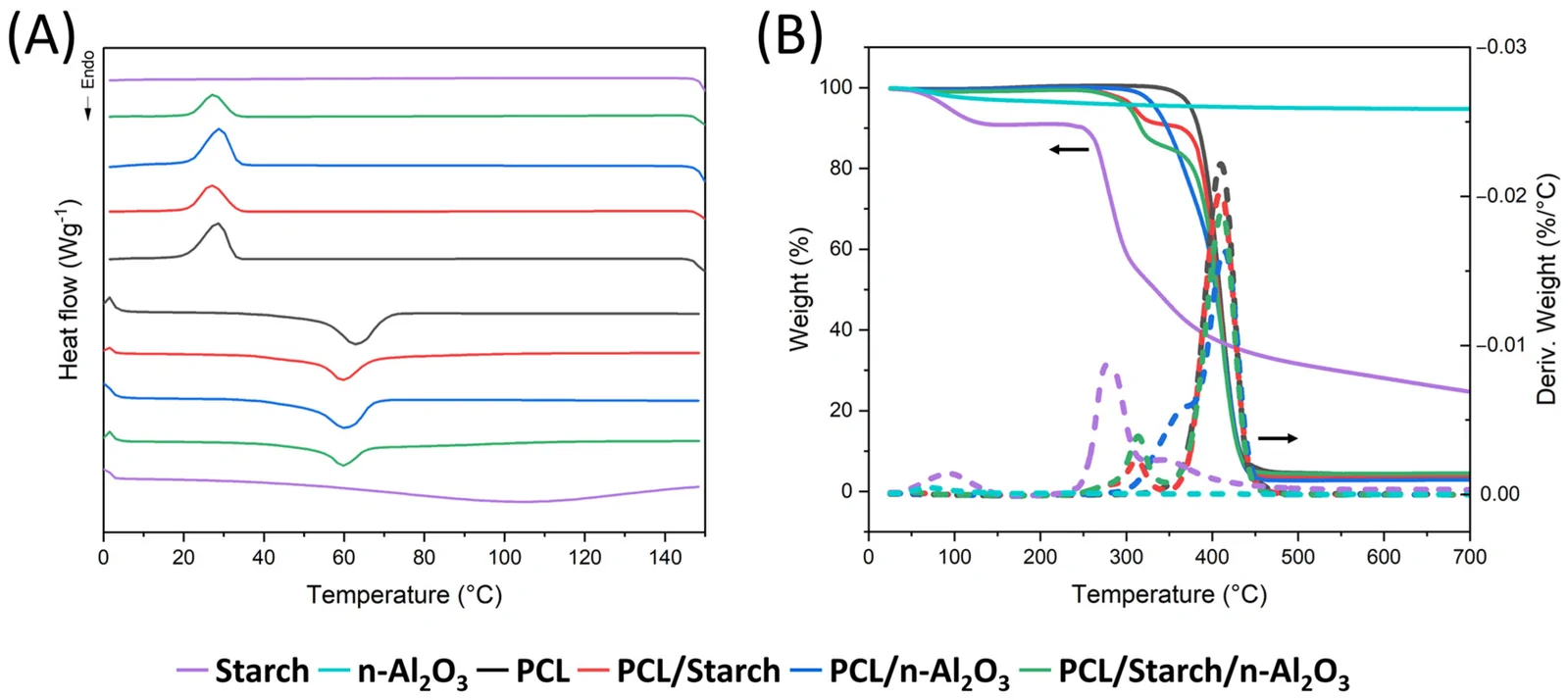
(**A**) DSC, (**B**) TGA and DTGA analysis for electrospun fibers and starch, PCL, PCL/n-Al_2_O_3_, PCL/starch and PCL/starch/n-Al_2_O_3_.

To study the thermal behavior of materials based on electrospun fibers, differential scanning calorimetry (DSC) studies were conducted. Figure 5A shows the results obtained for the first heating and cooling cycle and Table 2 shows the crystallization temperature (Tc), melting temperature (Tm), enthalpy of fusion (ΔH_f_) and crystallinity percent (Xc). The calorimetric profiles of PCL show two clearly distinguishable processes; endothermic melting at 63 °C and exothermic crystallization at 29 °C. This is in accordance with previous reports [66]. Furthermore, fibers containing starch and/or Al_2_O_3_ display a calorimetric profile similar to that of PCL fibers.

The first effect observed is that the incorporation of n-Al_2_O_3_ or starch into PCL fibers leads to a slight decrease in the crystalline percent of PCL/starch/n-Al_2_O_3_. Mina Hernandez reported that the relative crystallinity of PCL in thermoplastic starch and PCL blends [67], obtained by extrusion, decreases as the starch content in the blend increases. Since starch and PCL are immiscible, phase separation and low interaction between both are expected. In this way, starch can reduce crystallinity by inhibiting the movement of polymer chains required for crystallization. The crystallinity percent of PCL in PCL/starch/n-Al_2_O_3_ is lower than those in PCL/starch fibers due to the presence of n-Al_2_O_3_. Similarly, the addition of nanoparticles can also constrain the mobility of polymer chains, reducing crystallinity. By DRX analysis, Sharma and Pathak demonstrated that the PCL crystallinity in PCL/TiO_2_-nanoparticle composites decreases as TiO_2_ content increases [68]. In addition, the lower crystallinity percentage of PCL/starch/n-Al_2_O_3_ fibers can also be attributed to the confinement effects produced by the smaller diameter of these fibers. [65]. Another result is the decrease in the melting temperature of fibers PCL/starch/n-Al_2_O_3_ compared with neat PCL. In general, the reduction in melting temperature is attributed to the formation of less perfect crystalline structures [69].

The TGA thermograms are shown in Figure 5B, and the main parameters extracted from the TGA and DTGA curves are summarized in Table 2. Neat PCL exhibited the highest thermal stability, with a T_10%_ of 384 °C and a single major degradation peak at 410 °C, consistent with previous reports describing the one-step thermal decomposition of PCL [70]. In contrast, the incorporation of starch resulted in an earlier onset of degradation, as reflected by the reduced T_10%_ values, and introduced a clear two-step degradation profile for the PCL/starch (312 °C; 410 °C) and PCL/starch/n-Al_2_O_3_ (314 °C; 411 °C) scaffolds. The first weight-loss event can be attributed to the lower intrinsic thermal stability of starch, which typically decomposes in the 280–320 °C range, whereas the second step corresponds to the degradation of the PCL matrix [70]. The final n-Al_2_O_3_ content in the electrospun mats was estimated from the residual mass obtained by TGA and corresponded to 3 wt% of the total mat mass.

The incorporation of n-Al_2_O_3_ in the composite scaffolds further decreased the T_10%_ values (348 °C for PCL/n-Al_2_O_3_ and 317 °C for PCL/starch/n-Al_2_O_3_), indicating an earlier onset of mass loss. Nevertheless, the main degradation peak associated with PCL remained essentially unchanged (414 °C and 411 °C, respectively). Overall, these results suggest that the nanoparticles mainly affect the initial degradation stage, likely by promoting early thermal scission events, while having a limited impact on the principal decomposition of the PCL backbone. Similar trends have been reported for polymer matrices containing metal oxide nanoparticles, where additives such as MgO reduce the degradation onset temperature [71]. This effect has been attributed to surface hydroxyl groups acting as Brønsted acidic sites, which can catalyze chain cleavage and accelerate polymer degradation [72].

#### 2.2.4. Mechanical Properties

**Figure 6:**
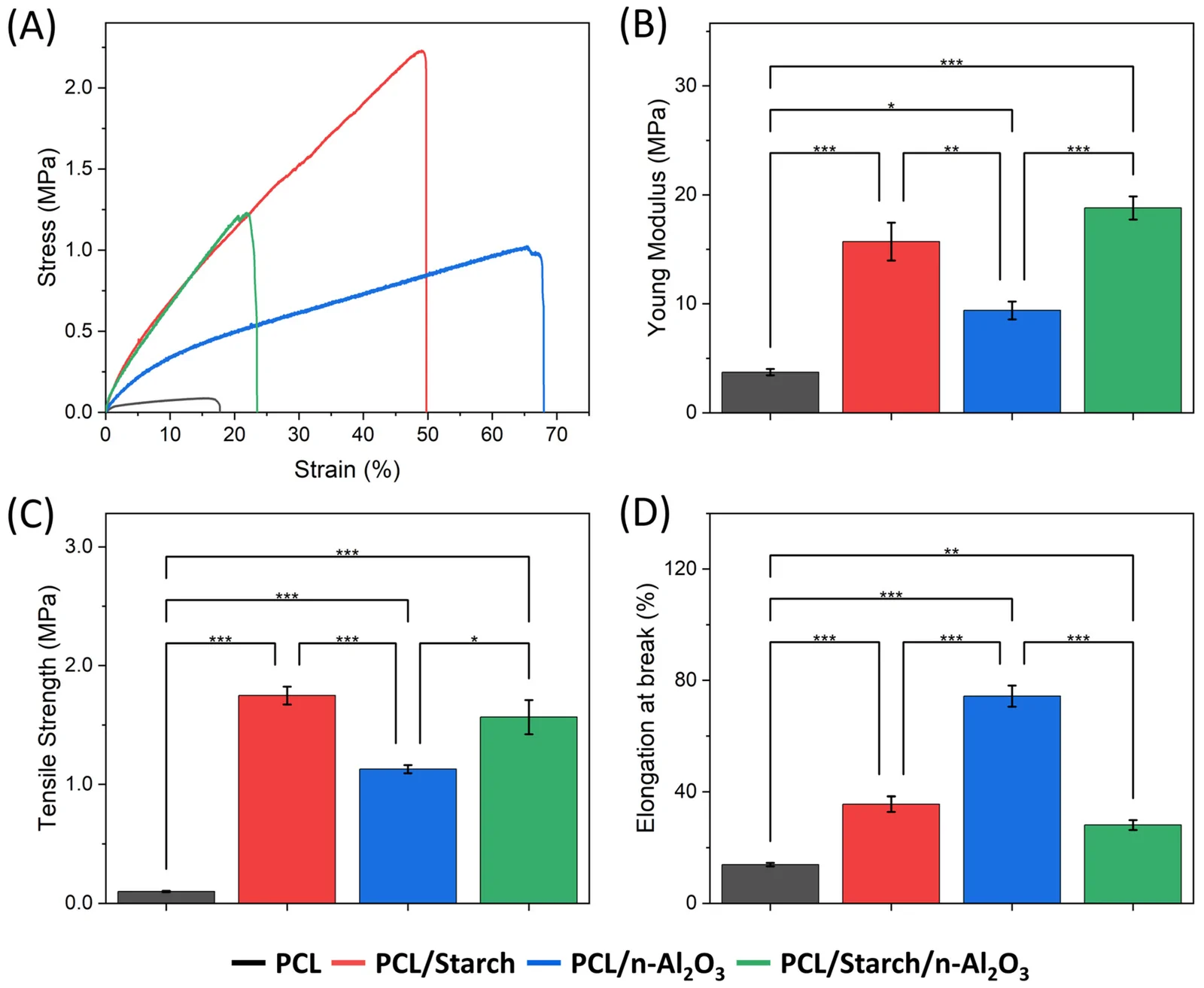
Tensile properties of electrospun PCL-based composite fibers, including PCL, PCL/starch, PCL/n-Al_2_O_3_, and PCL/starch/n-Al_2_O_3_ mats. (**A**) Stress–strain curves and comparison of (**B**) Young’s modulus, (**C**) tensile strength, and (**D**) elongation at break. Data are presented as the mean ± SD (*n* = 5 independent samples). Statistical differences were assessed by one-way ANOVA followed by Bonferroni’s multiple-comparison test. *p* < 0.05 (*), *p* < 0.01 (**), *p* < 0.001 (***).

The mechanical properties of the materials based on electrospun polymeric fibers were evaluated by tensile tests (Figure 6). Notably, the PCL/n-Al_2_O_3_ scaffold exhibited substantial enhancements in both Young’s modulus and elongation at break, which increased by 157% and 469%, respectively, compared with neat PCL. Usually, in composites, nanoparticles act as reinforcing agents. Dong et al. prepared electrospun PCL membranes incorporating Al_2_O_3_ nanowhiskers and reported that the addition of these nanowhiskers increased the Young’s modulus from approximately 2 MPa for neat PCL fibers to a maximum of about 16 MPa at 10 wt% Al_2_O_3_ [45]. Lamastra et al. reported that incorporating n-Al_2_O_3_ into electrospun poly(vinyl alcohol) (PVA) mats markedly enhanced their mechanical stiffness [73]. The Young’s modulus, which was ca. 90 MPa for neat PVA, rose to about 280 MPa and 350 MPa when 2 wt% and 9 wt% n-Al_2_O_3_ were added, respectively. The incorporation of nanoparticles increases the rigidity of the material by restricting the mobility of the polymer chains near the surface of the nanoparticles.

Regarding elongation at break, fibers with smaller diameters exhibit a higher degree of molecular orientation along the fiber’s axis, leading to lower values of elongation at break. Sauter et al. produced electrospun polyurethane meshes and found that fiber diameter plays a key role in molecular alignment, shape-memory performance, and mechanical properties [74]. As the diameter decreases, spatial confinement within the fibers promotes greater molecular orientation along the orientation of the fiber, while a decrease in the fiber diameter causes a reduction in elongation at break. Young’s modulus remained relatively constant across the examined diameters (2.3, 1.0, 0.3, and 0.08 μm); however, the elongation at break was highly diameter dependent. Fibers with a diameter of 2.3 μm reached an elongation at break of ca. 390%, whereas progressive diameter reduction resulted in a decreasing, reaching ca. 85% for fibers of ca. 0.08 μm. Lim et al. obtained electrospun PCL fibers with different diameters (345 nm, 540 nm, and 930 nm) [75]. Smaller fiber diameters were also associated with lower elongation at break. This behavior was attributed to the densely packed aligned lamellae and fibrillar structures within the fibers of smaller diameters. Molecular orientation produces more orderly and rigid structures; as a result, polymers lose ductility and show less deformation before failure. According to our results, the enhancement in Young’s modulus can be attributed to the reinforcing effect of the nanoparticles, while the greater diameter of the PCL/n-Al_2_O_3_ fibers, relative to fibers composed solely of PCL, explains the increased elongation at break exhibited by the PCL/n-Al_2_O_3_ fibers.

Compared with neat PCL, the PCL/starch fibers showed a marked improvement in mechanical performance, with a 321% increase in Young’s modulus and a 155% increase in elongation at break. This behavior is consistent with the rigid nature of starch, which can act as a reinforcing phase and increase the stiffness of PCL-based composites [76]. Similar trends have been reported for sago starch/PCL blends, where starch incorporation increases Young’s modulus, although often at the expense of ductility and tensile strength [77]. In our case, the simultaneous increase in elongation at break may be partially associated with morphological effects, such as the larger fiber diameter observed for PCL/starch fibers. In addition, starch solubilization in formic acid may promote esterification to form starch formate [30], which could enhance its affinity for PCL and improve interfacial stress transfer, contributing to the overall mechanical reinforcement.

Finally, the incorporation of n-Al_2_O_3_ into PCL/starch systems leads to an increase of 404% in Young’s modulus and a 102% in elongation at break compared to neat PCL. This additional stiffening effect can be attributed to the combined reinforcing contributions of starch and n-Al_2_O_3_, resulting in higher rigidity.

The mechanical properties of wound-healing materials play a crucial role in determining their performance and effectiveness. To support cell proliferation and facilitate proper tissue regeneration, these materials should closely match the mechanical properties of human skin [78,79]. The mechanical properties of the skin exhibit considerable variability due to its highly anisotropic nature and the distinct mechanical stresses to which it is subjected across different anatomical locations [79,80]. Consequently, its reported mechanical properties span a broad range, with Young’s modulus between 15 and 150 MPa, ultimate tensile strength between 1 and 32 MPa, and elongation at break between 35 and 115% [81]. Although the elongation at break of the PCL/starch/n-Al_2_O_3_ fibers (ca. 30%) is lower than the values reported for human skin, their Young’s modulus (ca. 18 MPa) and ultimate tensile strength (ca. 3 MPa) fall within the ranges reported for human skin, suggesting that the material possesses mechanical properties suitable for wound-healing applications. Thus, in addition to exhibiting suitable mechanical properties, these fibers are expected to display enhanced hydrophilicity and antimicrobial activity as a result of the incorporation of starch and n-Al_2_O_3_.

#### 2.2.5. Water Absorption and Weight Loss

**Figure 7:**
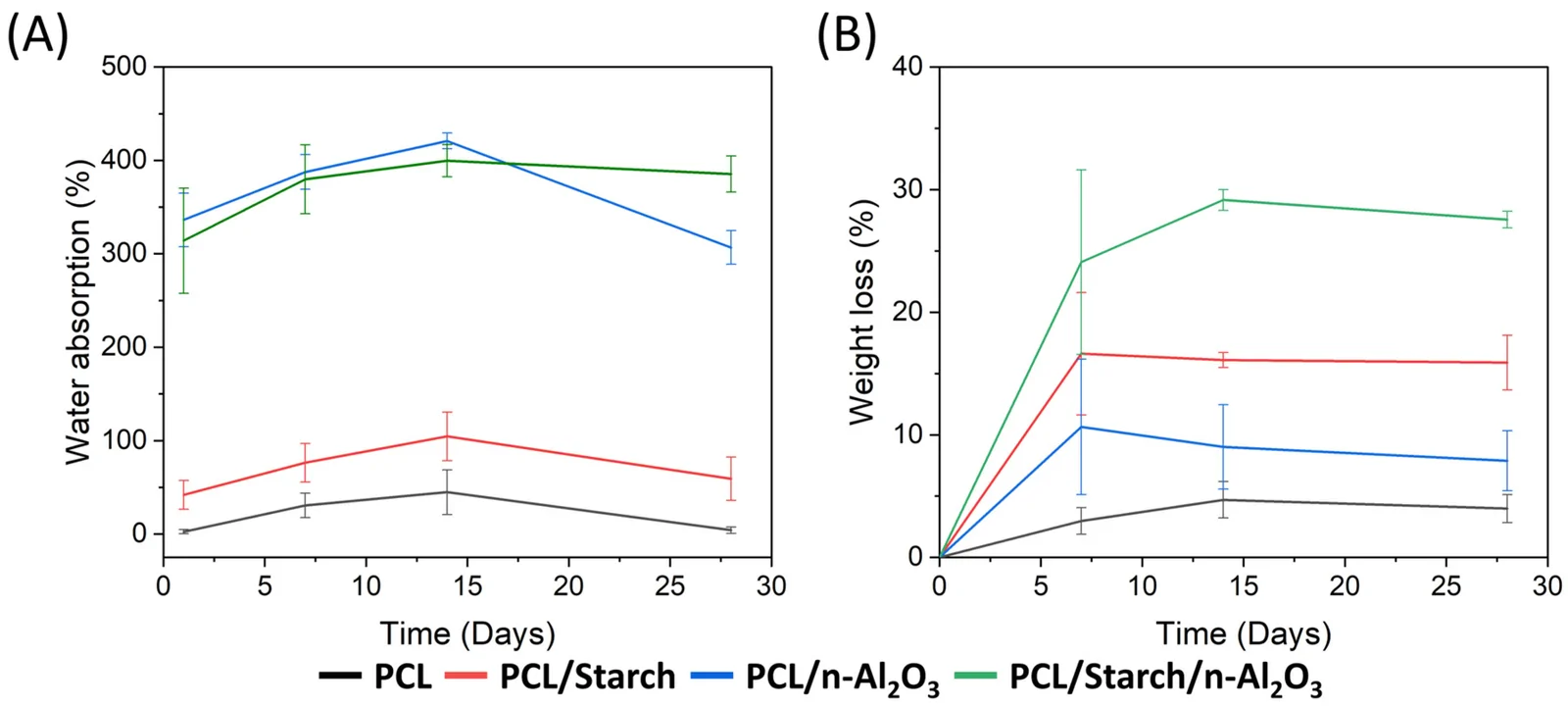
(**A**) Water absorption and (**B**) weight loss behavior of electrospun PCL, PCL/starch, PCL/n-Al_2_O_3_, and PCL/starch/n-Al_2_O_3_ fibers (during 28 days of immersion in PBS at 37 °C). Data are presented as the mean ± SD (*n* = 3 independent samples).

The hydrophilicity of fibers and their weight loss behavior under physiological conditions are critical parameters when assessing the suitability of these materials for biological applications. Hydrophilicity, typically determined through water absorption assays, plays a key role in wound healing materials by enabling efficient uptake of wound exudates [17]. Meanwhile, weight loss under physiological conditions, commonly evaluated by measuring mass loss or solubility in phosphate-buffered saline (PBS), is particularly important for tissue regeneration, as the scaffold must provide temporary structural support while facilitating new tissue formation [82]. Figure 7 shows water absorption (Figure 7A) and weight loss (Figure 7B) of the electrospun fibers during immersion in PBS for 28 days. Water uptake occurred within the first 24 h, followed by only moderate variations up to day 28. No significant time-dependent differences were observed for any formulation, indicating that scaffold composition had a greater influence on water absorption than immersion time.

As expected, PCL fibers exhibited low hydrophilicity, with water absorption of approximately 4.2% at 24 h. Furthermore, among the fibers studied, PCL fibers exhibited the lowest degradation rates. PCL degradation initially involves water absorption and subsequent breakdown of the polymer chains through hydrolysis of the ester groups. The hydrophobic nature of the long aliphatic chains of PCL limits its water absorption capacity and therefore its degradation rate [16]. It is important to note that the rate of PCL degradation is influenced by the arrangement of its molecular chains. In the highly ordered crystalline regions, water penetration is limited, which slows down hydrolytic degradation. In contrast, amorphous regions degrade more rapidly, as water can penetrate these areas more easily [83].

Due to the hydrophilic nature of starch and Al_2_O_3_, their incorporation enhanced the water absorption of the electrospun fibers compared with neat PCL. In particular, PCL/n-Al_2_O_3_ and PCL/starch/n-Al_2_O_3_ exhibited the highest water absorption, reaching approximately 300–400%. Augustine et al. reported that embedding ZnO nanoparticles within electrospun PCL fibers enhances their hydrophilicity, as evidenced by reduced contact angles, and also increases water uptake and degradation rates [84]. While these effects were primarily attributed to the improved hydrophilic nature of the composites, the authors suggested that variations in the surface-to-volume ratio might also influence both water absorption and weight loss behavior. Similarly, Giri Dev and Hemamalini observed that incorporating starch into PCL (1:1 ratio) resulted in an approximately 1200% increase in water absorption capacity compared with pure PCL fibers [30]. In turn, amorphous starch is water-soluble [85]; therefore, its incorporation into electrospun PCL fibers not only promotes the hydrolytic degradation of PCL but also contributes directly to material weight loss through its dissolution in PBS. Accordingly, the starch-containing fibers showed the greatest weight loss during the assay (Figure 7B). The higher weight loss of PCL/starch/n-Al_2_O_3_ compared with PCL/starch can be ascribed to three main factors: (i) increased water uptake by the PCL/starch/n-Al_2_O_3_ composite fibers, (ii) slight reduced crystallinity of the PCL phase within these fibers, and (iii) smaller average diameters of fibers [86].

### 2.3. Cell Viability Assay

**Figure 8:**
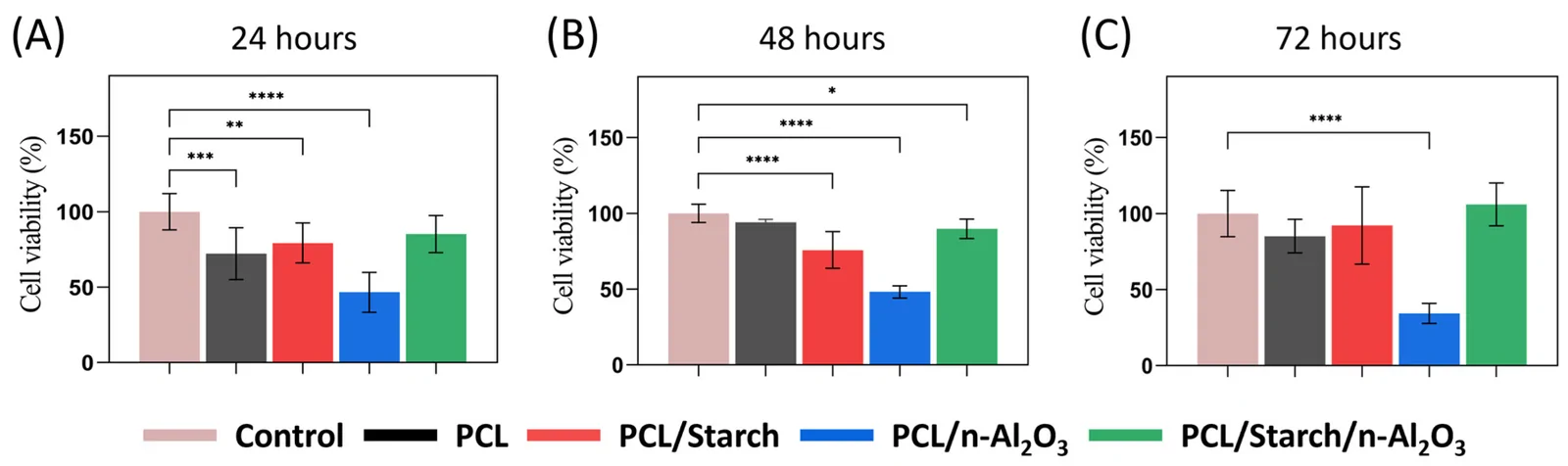
Cell viability test on NIH-3T3 cells cultured on electrospun PCL, PCL/starch, PCL/n-Al_2_O_3_, and PCL/starch/n-Al_2_O_3_ scaffolds. (**A**) 24 h, (**B**) 48 h, (**C**) 72 h. Data are presented as mean ± SD; *p* < 0.05 (*), *p* < 0.01 (**), *p* < 0.001 (***), *p* < 0.0001 (****) using the Bonferroni test (*n* = 3 independent samples).

The cytocompatibility of the electrospun scaffold extracts was evaluated using NIH-3T3 fibroblasts following the indirect extract method described in ISO 10993-5 [87]. As shown in Figure 8, cell viability exhibited a time-dependent response depending on the scaffold composition. The control group maintained approximately 100% viability throughout the study, whereas all scaffold extracts except PCL/n-Al_2_O_3_ preserved cell viability above the 70% cytotoxicity threshold established by ISO 10993-5.

After 24 h of exposure (Figure 8A), extracts from PCL, PCL/starch, and PCL/starch/n-Al_2_O_3_ maintained cell viabilities close to the control (approximately 94–98%), with no evidence of cytotoxicity. In contrast, the PCL/n-Al_2_O_3_ extract significantly reduced cell viability to approximately 75% (**** *p* < 0.0001 vs. control), although this value remained above the ISO cytotoxicity limit.

At 48 h (Figure 8B), the differences among formulations became more evident. Cell viability remained high for PCL (≈93%) and PCL/starch (≈87%), whereas the ternary PCL/starch/n-Al_2_O_3_ scaffold maintained a viability of approximately 83%. Conversely, the PCL/n-Al_2_O_3_ extract induced a marked reduction in metabolic activity (≈62%), falling below the 70% viability threshold defined by ISO 10993-5, indicating a cytotoxic response. Statistical analysis confirmed significant differences between PCL/n-Al_2_O_3_ and the control (**** *p* < 0.0001), as well as with PCL/starch (* *p* < 0.05) and PCL/starch/n-Al_2_O_3_ (** *p* < 0.01).

After 72 h of incubation (Figure 8C), the same trend was maintained. Extracts from PCL, PCL/starch, and PCL/starch/n-Al_2_O_3_ continued to exhibit high cell viability (approximately 82–95%), demonstrating sustained cytocompatibility over prolonged exposure. In contrast, the PCL/Al_2_O_3_ extract further decreased cell viability to approximately 48% (**** *p* < 0.0001 vs. control), indicating that the biological effect associated with this formulation increased with incubation time.

The progressive reduction in cell viability observed for the PCL/n-Al_2_O_3_ extracts suggests that extractable components released from the scaffold adversely affected fibroblast metabolic activity. According to ISO 10993-5, indirect cytotoxicity assays evaluate the biological response to substances released from biomaterials rather than direct cell–material interactions. Therefore, the observed reduction in viability is likely associated with soluble or suspended species present in the extract. Previous studies have demonstrated that the biological response to alumina nanoparticles is strongly influenced by their physicochemical characteristics, including particle size, concentration, surface properties, and aggregation state, all of which affect nanoparticle–cell interactions and cellular uptake [88,89,90]. Since nanoparticle release was not quantified in the present study, the precise mechanism responsible for the reduced viability cannot be established. Further investigations evaluating the release profile of nanoparticles and other extractable components from the electrospun scaffolds are required to clarify the origin of this cytotoxic response.

Previous studies on hMSC 5077-GFP cells (hrGFP-transduced human bone marrow stromal cells) reported that PCL/Al_2_O_3_ electrospun fibers exhibited good biocompatibility and supported cell growth, maintaining cell viability values of 88% at 24 h and 73% at 48 h [45]. While there is not complete consensus regarding which specific characteristics of n-Al_2_O_3_ induce toxicity, the literature indicates that larger particles sizes [91], rod-like morphologies [92], the η-Al_2_O_3_ crystallographic phase [37], and higher concentrations can result in cytotoxic effects [38].

Interestingly, incorporating starch into the PCL/n-Al_2_O_3_ system prevented the cytotoxic response observed for the binary composite. Throughout the 72 h evaluation period, extracts from the PCL/starch/n-Al_2_O_3_ scaffolds consistently maintained cell viability above 80%, indicating that starch effectively mitigated the adverse biological effects associated with the PCL/n-Al_2_O_3_ formulation. A plausible explanation is that starch promotes a more homogeneous distribution of alumina nanoparticles within the polymeric matrix, thereby reducing nanoparticle aggregation and limiting the release of extractable particulate species into the extraction medium. In addition, the hydrophilic nature of starch may stabilize the polymer network, reducing the diffusion of potentially bioactive components. Similar improvements in cytocompatibility have been reported in polymer–ceramic composites containing natural polysaccharides, where starch enhanced filler dispersion and reduced adverse cellular responses [93]. The improved performance of the starch into the electrospun fibers underscores the importance of surface wettability in modulating cellular responses. Starch, being hydrophilic, facilitates fibroblast adhesion and polymer proliferation [54,56]. García et al. evaluated electrospun PCL/starch/CaO fibers using the MC3T3-E1 cell line, finding no cytotoxic response. Additionally, in vivo results indicated that the hydrophilicity of starch enhanced the scaffold [54].

Overall, these results demonstrate that all scaffold extracts except PCL/n-Al_2_O_3_ satisfied the cytocompatibility requirements established by ISO 10993-5. The ternary PCL/starch/n-Al_2_O_3_ formulation exhibited sustained cell viability throughout the study, suggesting that starch plays an essential role in modulating the biological response of alumina-containing electrospun fibers and supporting their potential application as wound dressing materials.

### 2.4. Hemolysis Assay

**Figure 9:**
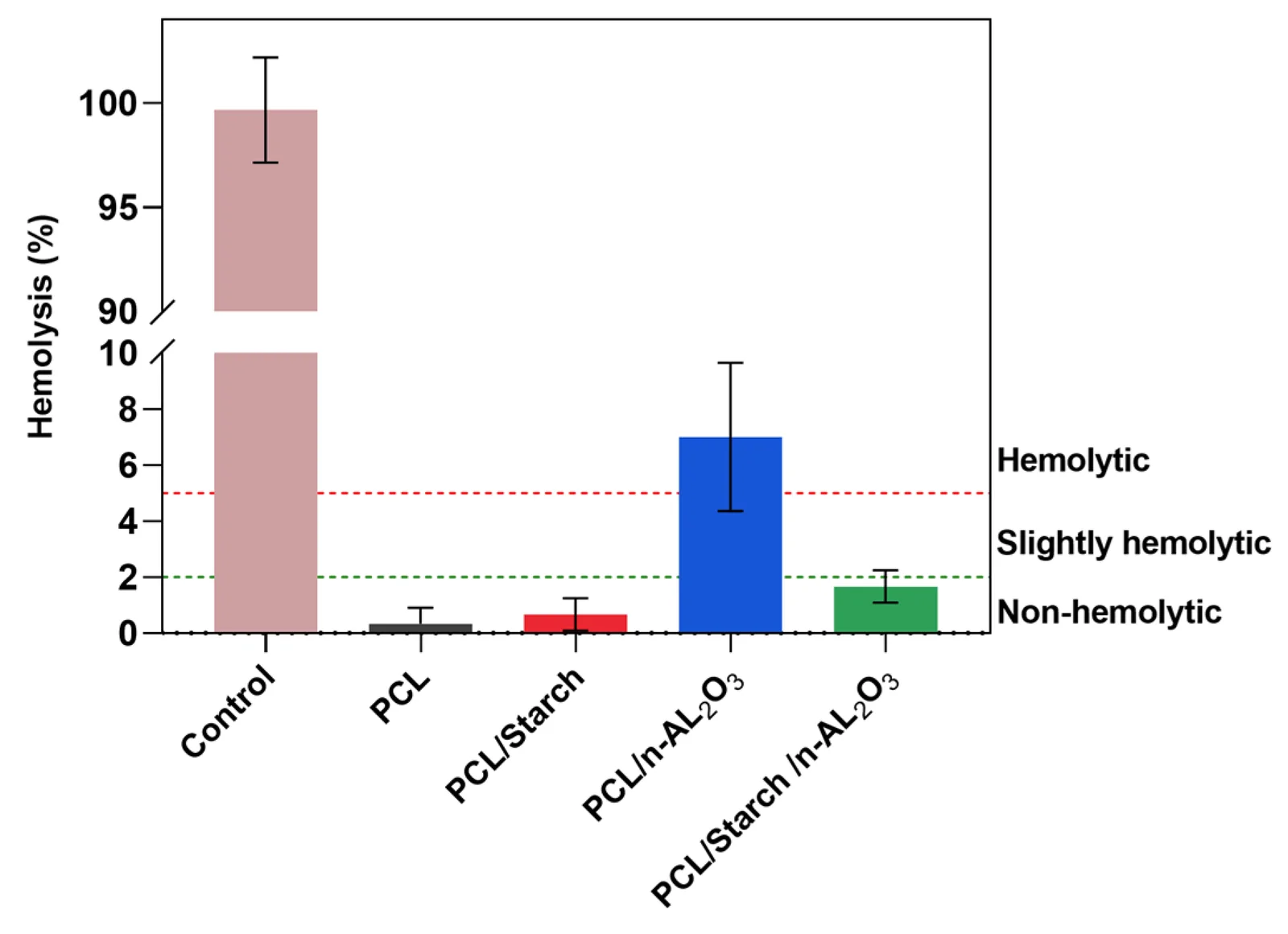
Hemolysis assessment of electrospun PCL, PCL/starch, PCL/n-Al_2_O_3_, and PCL/starch/n-Al_2_O_3_ scaffolds. Hemolytic activity was determined according to the ASTM F756-17 standard by measuring the release of hemoglobin at 540 nm after incubation with mouse erythrocytes. Data are presented as mean ± SD (*n* = 3 independent samples).

Hemocompatibility is a critical requirement for biomaterials intended for wound healing applications, as these materials are expected to establish direct contact with blood immediately after implantation or placement on injured tissue. According to the ASTM F756-17 standard [94], materials exhibiting hemolysis values below 2% are classified as non-hemolytic, values between 2 and 5% as slightly hemolytic, and those exceeding 5% as hemolytic (ASTM F756-17 [94]). In the present study (Figure 9), PCL, PCL/starch, and PCL/starch/n-Al_2_O_3_ scaffolds exhibited hemolysis values below the non-hemolytic threshold, whereas the PCL/n-Al_2_O_3_ scaffold induced approximately 7% hemolysis, exceeding the ASTM limit for blood-compatible biomaterials. These findings demonstrate that the incorporation of starch effectively mitigated the hemolytic effect associated with alumina nanoparticles while preserving the overall hemocompatibility of the composite [95].

The excellent hemocompatibility observed for neat PCL is consistent with previous reports describing this polyester as a highly biocompatible material with minimal erythrocyte damage. Owing to its chemical stability, low degradation rate, and favorable surface characteristics, PCL generally exhibits limited interaction with erythrocyte membranes, resulting in negligible hemoglobin release during direct blood-contact assays [96]. These properties have contributed to its widespread use in electrospun wound dressings and tissue engineering scaffolds, where blood compatibility is essential during the early stages of tissue repair.

Conversely, the incorporation of Al_2_O_3_ nanoparticles in the absence of starch significantly increased hemolytic activity. Nanoparticle-mediated hemolysis has been extensively associated with the physicochemical characteristics of inorganic nanomaterials, including particle size, surface charge, aggregation state, and surface energy, all of which influence their interaction with erythrocyte membranes [97]. Alumina nanoparticles possess a relatively high surface energy and may adsorb plasma proteins or directly interact with the phospholipid bilayer of red blood cells, promoting membrane destabilization, oxidative stress, and ultimately hemoglobin leakage. Similar mechanisms have been reported for several ceramic nanoparticles, where increased surface reactivity correlates with greater erythrocyte membrane disruption [97].

Despite this, the simultaneous incorporation of starch markedly reduced hemolysis from approximately 7% to values below 2%, indicating that starch played a protective role against erythrocyte damage. This improvement is likely associated with the intrinsic hydrophilicity of starch, which increases surface hydration and enhances the blood compatibility of polymeric biomaterials. Hydrophilic polysaccharides have been widely reported to promote the formation of hydrated interfaces that reduce nonspecific protein adsorption and minimize unfavorable blood–material interactions, thereby improving hemocompatibility [98]. In addition, starch may contribute to a more homogeneous distribution of Al_2_O_3_ nanoparticles within the PCL matrix, decreasing nanoparticle aggregation and reducing the direct exposure of highly reactive ceramic surfaces to erythrocytes. Similar strategies employing natural polysaccharides have been shown to improve the biological performance and blood compatibility of hybrid wound dressings by combining the mechanical advantages of inorganic fillers with the excellent biocompatibility of hydrophilic biopolymers [99].

From a wound healing perspective, these findings are particularly relevant because an ideal wound dressing should not induce erythrocyte damage during the initial hemostatic phase. Excessive hemolysis may increase local inflammatory responses through the release of free hemoglobin and iron, potentially delaying tissue repair and impairing the regenerative microenvironment. Therefore, the non-hemolytic behavior exhibited by the PCL/starch/n-Al_2_O_3_ electrospun fibers suggests that the addition of starch effectively compensates for the adverse blood interactions associated with alumina nanoparticles while maintaining the potential mechanical reinforcement provided by the ceramic phase. Overall, these results support the suitability of the PCL/starch/n-Al_2_O_3_ scaffold as a promising blood-compatible material for wound dressing and skin tissue regeneration applications.

### 2.5. Cell Migration Assay

**Figure 10:**
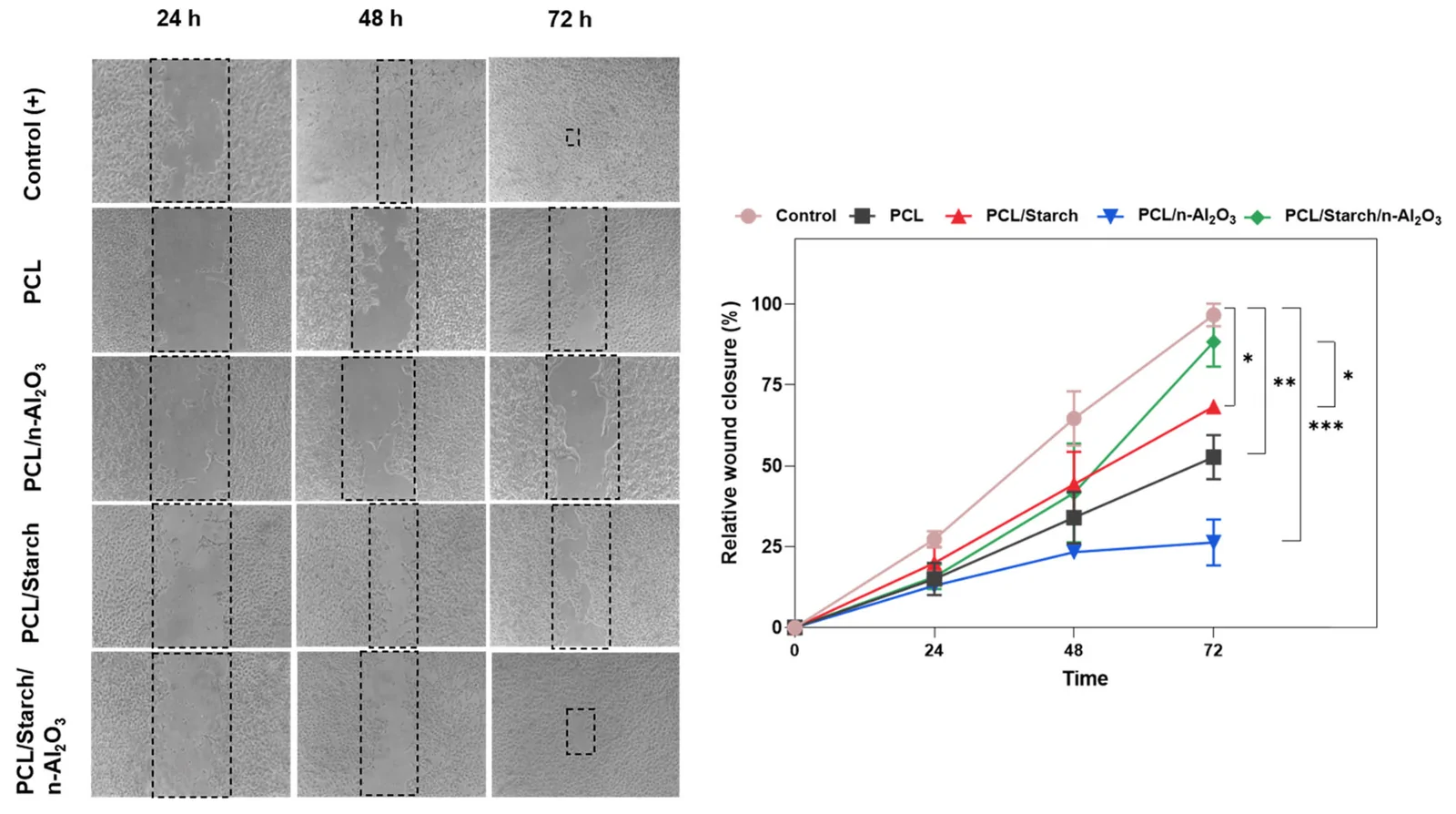
Representative phase-contrast micrographs and quantitative analysis of the scratch wound healing assay performed using NIH-3T3 fibroblasts exposed to extracts obtained from electrospun PCL-based scaffolds. Images were acquired at 24, 48 and 72 h after scratch formation. The dashed lines indicate the wound margins, whereas the dashed squares represent complete wound closure. The graph shows the percentage of relative wound closure over time. Data are expressed as mean ± SD (n = 3 independent samples). Statistical significance was determined by one-way ANOVA followed by Bonferroni’s multiple comparison test (* *p* < 0.05, ** *p* < 0.01, *** *p* < 0.001).

The scratch wound healing assay (Figure 10) demonstrated that extracts from the electrospun scaffolds differentially modulated fibroblast migration over time. At 24 h, all experimental groups exhibited partial wound closure, with no marked differences among treatments. However, differences became evident after 48 h, where extracts from the PCL/starch and PCL/starch/n-Al_2_O_3_ scaffolds significantly enhanced cell migration compared with the PCL/n-Al_2_O_3_ group.

After 72 h, the positive control showed nearly complete wound closure (~97%), while the PCL/starch/n-Al_2_O_3_ extract exhibited the highest migratory response among the scaffold-treated groups (~88%), approaching the control. The PCL/starch extract also promoted substantial wound closure (~68%), whereas the PCL extract induced only moderate migration (~52%). In contrast, cells exposed to the PCL/n-Al_2_O_3_ extract displayed the lowest wound closure (~26%), remaining significantly lower than all other groups (*p* < 0.05–0.001).

Overall, these findings indicate that incorporation of starch effectively counteracted the inhibitory effect associated with n-Al_2_O_3_, promoting fibroblast migration and suggesting improved bioactivity of the hybrid scaffold. The enhanced migratory response observed for the PCL/starch/n-Al_2_O_3_ formulation supports its potential to stimulate cellular events involved in the proliferative phase of wound healing [56,89,93].

### 2.6. Protein Expression Analysis

**Figure 11:**
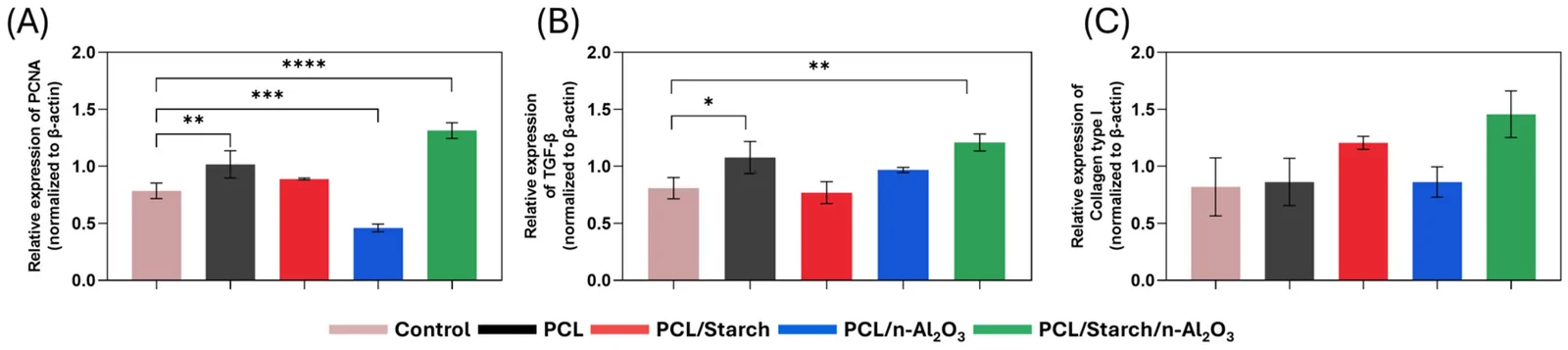
Relative expression of wound healing-related proteins in NIH-3T3 fibroblasts after five days of exposure to extracts obtained from electrospun PCL-based scaffolds. (**A**) PCNA, (**B**) TGF-β1 and (**C**) Collagen type I protein expression quantified by indirect ELISA and normalized to β-actin. Data are expressed as mean ± SD (*n* = 3 independent samples). Statistical significance was determined by one-way ANOVA followed by Bonferroni’s multiple comparison test (* *p* < 0.05, ** *p* < 0.01, *** *p* < 0.001, **** *p* < 0.0001).

After five days of exposure, scaffold extracts differentially modulated the expression of proteins associated with fibroblast proliferation and extracellular matrix formation (Figure 11). PCNA expression was significantly enhanced in cells treated with the PCL/starch/n-Al_2_O_3_ extract, reaching the highest level among all scaffold groups (approximately 1.3-fold), whereas the PCL/n-Al_2_O_3_ extract produced the lowest expression, significantly lower than the hybrid starch-containing scaffold. Intermediate values were observed for the PCL and PCL/starch extracts.

A similar trend was observed for TGF-β1, where the PCL/starch/n-Al_2_O_3_ extract induced the greatest protein expression, significantly exceeding both the control and PCL/starch groups. Conversely, PCL and PCL/n-Al_2_O_3_ promoted only moderate increases, while PCL/starch remained comparable to the control.

Although Collagen type I expression did not reach statistical significance, a clear tendency toward increased collagen production was observed in the PCL/starch/n-Al_2_O_3_ group, followed by the PCL/starch extract. In contrast, the PCL and PCL/n-Al_2_O_3_ extracts showed collagen levels similar to those of the untreated control.

The enhanced expression of PCNA and TGF-β1 observed in NIH-3T3 fibroblasts exposed to the PCL/starch/n-Al_2_O_3_ extract suggests that this formulation promotes cellular events associated with the proliferative phase of wound healing. PCNA is a well-established marker of DNA synthesis and cell proliferation; therefore, its upregulation indicates increased fibroblast proliferative activity, which is essential for granulation tissue formation and tissue regeneration. Likewise, TGF-β1 is one of the principal cytokines orchestrating wound repair by stimulating fibroblast proliferation, migration, differentiation into myofibroblasts, and extracellular matrix synthesis, including collagen deposition. The concomitant increase in both proteins agrees with the accelerated wound closure observed in the migration assay, indicating that soluble bioactive components released from the hybrid scaffold generate a microenvironment favorable for tissue repair. Although Collagen type I expression did not reach statistical significance, its upward trend in the PCL/starch/n-Al_2_O_3_ group may indicate the early activation of extracellular matrix synthesis, since collagen deposition generally increases progressively as the proliferative phase transitions into tissue remodeling [100,101].

### 2.7. Antibacterial Activity Test

**Figure 12:**
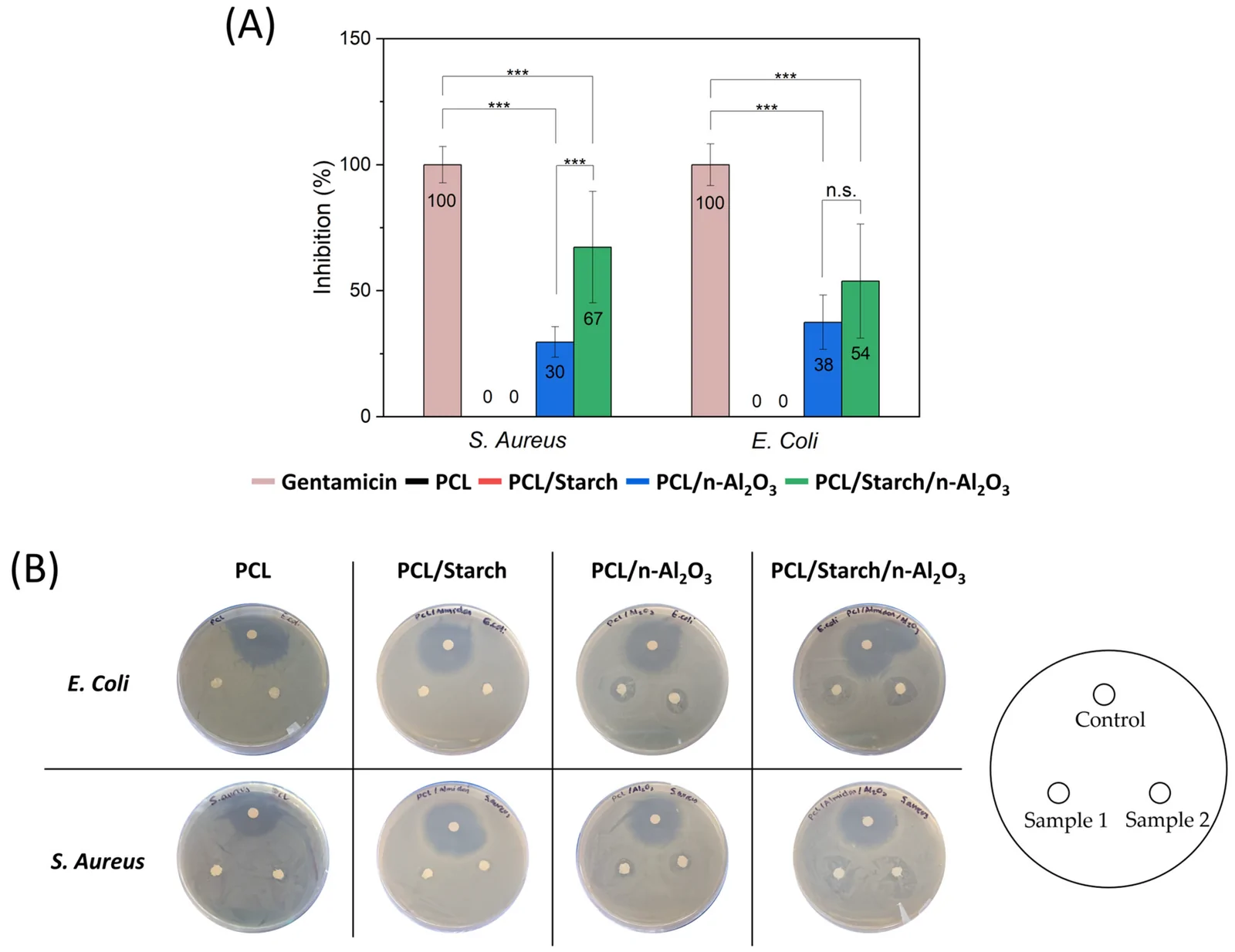
Inhibition activity of electrospun PCL, PCL/starch, PCL/n-Al_2_O_3_, and PCL/starch/n-Al_2_O_3_ scaffolds against *E. coli* and *S. aureus*. (**A**) Quantification of bacterial growth inhibition (%), (**B**) Representative agar disk-diffusion images and schematic representation of the sample distribution on the plates. Data are presented as mean ± SD; *p* < 0.001 (***), n.s. = no significant, using the Bonferroni test (*n* = 2).

Figure 12 shows the inhibition activity of each material. While PCL and PCL/starch did not show any activity against *S. aureus* and *E. coli*, the fibers with nanoparticles demonstrated to have inhibition against both bacteria. Lower inhibition potency compared to the control (Gentamicin) is observed, specifically 69% lower against *E. coli* and 64% against *S. aureus* for PLC/n-Al_2_O_3_, while in the ternary system, inhibition was 31% against *E. coli* and 50% against *S. aureus*. Al_2_O_3_ has been demonstrated to show some inhibition in bacterial growth, for example, fabric with deposited Al_2_O_3_ displayed inhibition against both cell cultures [102]. In a different study, nanowhiskers of Al_2_O_3_ showed an initial susceptibility to *E. coli* and *S. aureus*, but the cell culture regrew over time [33]; therefore, results suggest that initial inhibition can occur, delaying the possibility of infection in early stages thanks to this effect. The presence of starch in the ternary system enhances the inhibition of both cell cultures as starch improves nanoparticle distribution into the polymer and because its hydrophilic nature makes it dissolve faster, exposing more nanoparticles to the media [103]. It should be noted that the agar disk diffusion assay was used as an initial qualitative screening method to evaluate the antibacterial activity of the developed materials. Although appropriate for this purpose, additional antibacterial assays would provide a more comprehensive evaluation of their antibacterial performance and will be considered in future studies.

Different mechanisms govern antimicrobial activity. Authors had reported that the main mechanism is the electrostatic interaction between the surface of Al_2_O_3_ nanoparticles and the membrane or cell wall, which causes the loss of integrity of the cell wall following the penetration of nanoparticles and ROS species from the formation of aluminum cations [32]. Then, antimicrobial activity is correlated with the surface area of the nanoparticles; phases with very high surfaces, such as γ-Al_2_O_3_ and η-Al_2_O_3,_ may exhibit a strong antimicrobial response but may be considered potentially toxic for tissue engineering applications [32]. In our case, since n-Al_2_O_3_ is a combination of different crystalline phases, the surface area of 135.5 m^2^ g^−1^ is lower, and thus the negative effects are reduced.

## 3. Materials and Methods

### 3.1. Materials

Nanoparticle synthesis and scaffold fabrication: Polycaprolactone (PCL; average Mn 80,000), starch from potato, Triton^TM^ X-100 (laboratory grade), Aluminum isopropoxide (≥98%) and Pluronic F-127 were purchased from Sigma-Aldrich (St. Louis, MO, USA). Dichloromethane (DCM; ≥99.8% for analysis EMSURE ACS, ISO, Reag. Ph Eur), formic acid (FA; 98–100% for analysis EMSURE ACS, ISO, Reag. Ph Eur), nitric acid (HNO_3_ 65%, for analysis EMSURE Reag. Ph Eur, ISO), ethanol absolute (≥99.5% EMPARTA ACS) and water (suitable for LC/MS, LiChrosolv) were purchased from Merck (Darmstadt, Germany).

PBS solution: Water (LC-MS grade, LiChrosolv), sodium chloride (NaCl (≥99.5% for analysis EMSURE), potassium chloride (KCl ≥ 99.5% for analysis EMSURE), di-sodium hydrogen phosphate (Na_2_HPO_4_ ≥ 99.0% anhydrous for analysis EMSURE^®^ ACS, Reag. Ph Eur), potassium dihydrogen phosphate (KH_2_PO_4_ ≥ 99.5%) was purchased from Merck.

Cell viability and antimicrobial assays: Dulbecco’s Modified Eagle Medium (DMEM; Gibco, Thermo Fisher Scientific, Waltham, MA, USA; Cat. No. 11965-092). Bovine serum (FBS; Gibco, Thermo Fisher Scientific, Waltham, MA, USA; Cat. No. 16000-044). 1% antibiotic–antimycotic solution (A/A; Sigma-Aldrich; Cat. No. A5955). [3-(4,5-dimethylthiazol-2-yl)-5-(3-carboxymethoxyphenyl)-2-(4-sulfophenyl)-2H-tetrazolium] (MTS; Cayman Chemical, Ann Arbor, MI, USA; Cat. No. 30060). Phenazine methosulfate (PMS; Cayman Chemical, Ann Arbor, MI, USA; Cat. No. 14963). Gentamicin sulfate (Biological Industries, Sartorius, Cromwell, CT, USA, Cat. No. 41-503).

### 3.2. Synthesis of Aluminum Oxide Mesoporous Nanoparticles

Aluminum oxide mesoporous nanoparticles (n-Al_2_O_3_) were synthesized using a previously reported sol-gel method, as described below [52]. An amount of 0.75 g of Pluronic F-127 was dissolved in 40 mL of absolute ethanol, and the mixture was stirred until a transparent solution was achieved. Subsequently, 1.02 g of aluminum isopropoxide and 0.8 mL of nitric acid (65%) were added to the solution, which was stirred for an additional 12 h. To evaporate the solvent, the resulting solution was placed in an oven at 60 °C for 48 h. This step led to the formation of a pale-yellow solid, which was subsequently transferred to a muffle furnace and calcinated at 900 °C for 6 h, finally resulting in a white powder.

#### Characterization of Aluminum Oxide Mesoporous Nanoparticles

The structural characterization of the Al_2_O_3_ nanoparticles was conducted by X-ray diffraction analysis. Diffractograms were taken using a Bruker D8 ADVANCE diffractometer(Bruker AXS GmbH, Karlsruhe, Germany) in the range 20° < 2θ < 70° and CuKα radiation (λ = 1.5406 Å). The average crystallite size was estimated using the Scherrer equation (Equation (1))

(1) $$D=\frac{k\lambda }{\beta cos\left(\theta \right)}$$

where *D* is the average crystal size, *k* is a shape factor (0.9), β is the full width at half-maximum of the X-ray diffraction peak in radians, λ is the X-ray wavelength, and θ is the Bragg angle.

Morphology and size were analyzed using a Hitachi HT7700 (Hitachi High-Tech Corporation, Tokyo, Japan) transmission electron microscope. Prior to microscopy analysis the nanoparticles were sonicated in water/ethanol and deposited on a copper grid. The mesoporous surface area of nanoparticles was determined by nitrogen adsorption–desorption isotherms at 77 K using a Gemini VII 2390t Micromeritics (Micromeritics Instrument Corporation, Norcross, GA, USA). Degassing was performed with a temperature ramp to 363 K for 1 h followed by 623 K for 4 h. The apparent surface area was calculated using the Brunauer–Emmett–Teller (BET) method. Total pore volume was determined at p/p0 = 0.99 and, the average pore size was determined using the Barrett–Joyner–Halenda (BJH) method.

### 3.3. Preparation of Electrospinning Solutions

Three different electrospinning solutions were prepared: PCL, PCL/n-Al_2_O_3_ and PCL/starch. To prepare the PCL fed solution (FS-A), PCL was dissolved in DCM at a concentration of 10% (*w*/*v*) and stirred at 300 rpm for 24 h at room temperature. For the PCL/n-Al_2_O_3_ feed solution (FS-B), n-Al_2_O_3_ nanoparticles (0.6% *w*/*v*; corresponding to 6% in weight respect to PCL) were first dispersed in DCM by sonication for 30 min in the presence of Triton X-100 (2% *w*/*v*) to promote a homogeneous nanoparticle dispersion. Subsequently, PCL was added to a final concentration of 10% *w*/*v* and stirred for 24 h. The PCL/starch feed solution (FS-C) was obtained by mixing two separate solutions: PCL dissolved in DCM (10% *w*/*v*) and starch dissolved in FA (10% *w*/*v*). These individual solutions were then mixed in a 1:1 volume ratio to obtain an electrospinnable blend. Each solution was stirred at 300 rpm at room temperature for 24 h.

### 3.4. Electrospun Fibers Fabrication

The fibers were produced by electrospinning using a Tong Li Tech TL-01 (TongLi Tech, Changzhou, China) spinneret operated at room temperature in a side-by-side configuration. Two polymer solutions were independently delivered through separate flow channels connected to adjacent outlet needles. This arrangement allowed the droplets formed at the needle tips to merge before jet formation, promoting the combination of both solutions within a single electrospinning jet. Both solutions were pumped at a constant flow rate of 1 mL h^−1^. The applied voltage ranged from 16 to 18 kV. The resulting fibers were collected on a stainless-steel rotating drum (170 rpm) placed 11 cm from the tip of the 20 G needle. To ensure complete solvent removal, the obtained mats were stored in a desiccator for 48 h and subsequently refrigerated at 4 °C until characterization.

**Figure 13:**
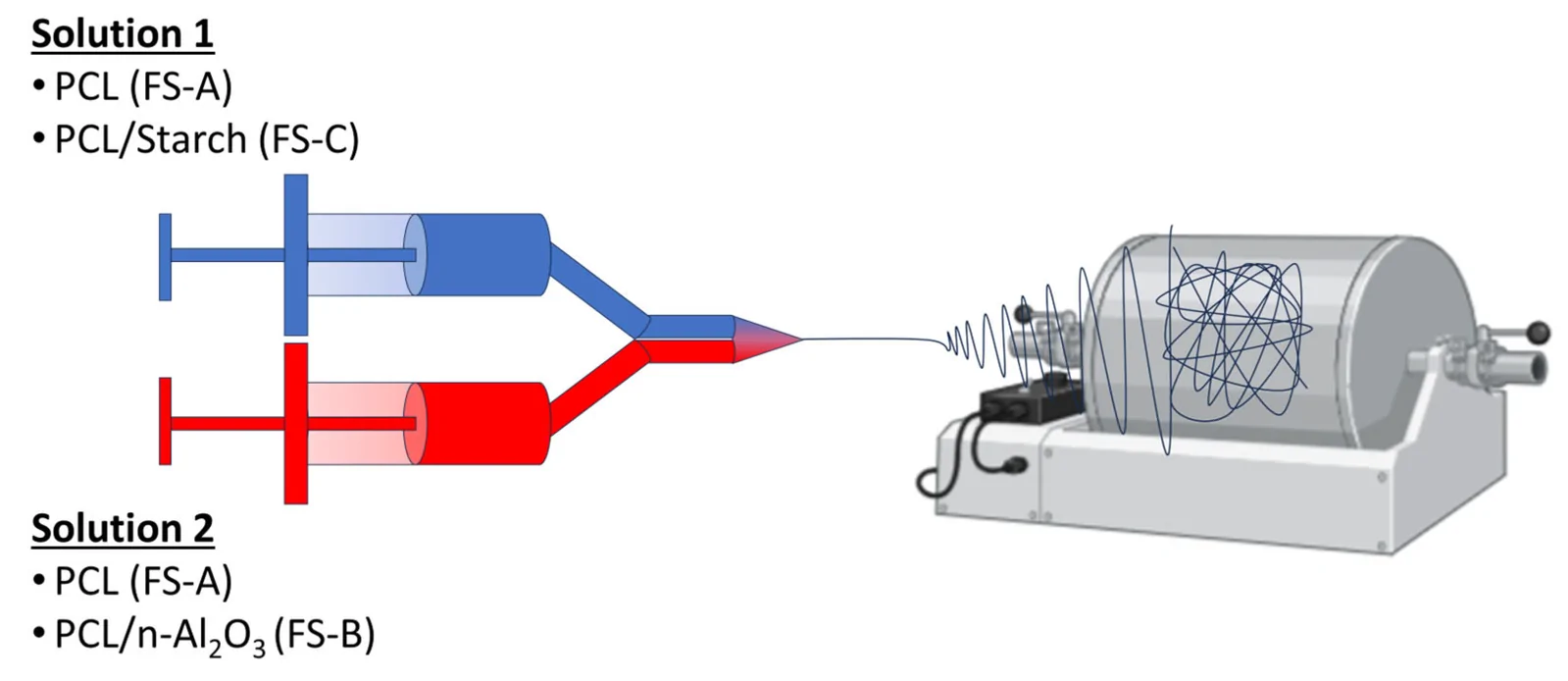
Schematic of a side-by-side electrospinning setup used to produce composite fibers from two independently fed solutions.

The composition of polymers and nanoparticles in the electrospun mats was controlled by the fed solutions delivered to each channel of the side-by-side setup as shown in Figure 13. Neat PCL mats were obtained by feeding a FS-A solution through both channels. Composite fibers were produced by replacing one of the channels with the corresponding formulation: FS-B (PCL/n-Al_2_O_3_) or FS-C (PCL/starch). For the ternary system (PCL/starch/n-Al_2_O_3_), FS-B and FS-C solutions were simultaneously loaded into separate channels (one per channel). This strategy enabled a direct comparison of all fiber mats under identical processing conditions. The expected compositions of the electrospun samples are summarized in Table 3.

### 3.5. Electrospun Fibers Characterization

#### 3.5.1. Morphological Characterization

The morphology of the electrospun fibers was analyzed by scanning electron microscopy (SEM) using a Zeiss EVO10 microscope (Carl Zeiss AG, Oberkochen, Germany). Prior to SEM analysis, the samples were coated with gold by using the Cressington 108 Auto Sputter Coater. Image analysis was conducted using Image-J software (version 1.53e, National Institutes of Health, Bethesda, MD, USA), Wayne Rasband, National Institute of Mental Health, NIH, Bethesda, MD, USA. For each sample, approximately 100 fibers were measured from 5 different SEM images and analyzed to evaluate fiber diameter distribution.

#### 3.5.2. Chemical Characterization

The chemical nature of the materials was analyzed using Fourier-transform infrared spectroscopy with attenuated total reflection (ATR-FTIR) on a PerkinElmer Spectrum Two instrument (PerkinElmer Inc., Waltham, MA, USA). FTIR spectra were taken in the range of 4000–450 cm^−1^ with a resolution of 4 cm^−1^, accumulation of 16 scans and a step increment of 1 cm^−1^.

#### 3.5.3. Crystal Phase Determination

The crystalline structure of the polymer within the electrospun fibers was characterized by X-ray diffraction (XRD) using a Bruker D8 ADVANCE diffractometer (Bruker AXS GmbH, Karlsruhe, Germany) equipped with Cu Kα radiation (λ = 1.5406 Å) and operated over a 2θ range of 2–80°. The average crystallite size was estimated by Scherrer equation (Equation (1))

#### 3.5.4. Thermal Analysis

Differential Scanning Calorimetry (DSC) measurements were carried out using a Mettler Toledo DSC1/500 instrument (Mettler-Toledo AG, Greifensee, Switzerland). Samples were heated from 0 °C to 150 °C in a nitrogen atmosphere at 10 °C/min, and then held at 150 °C for 10 min to stabilize, and finally cooled to 0 °C.

The crystallinity percent (Xc (%)) of PCL in the fibers was calculated using the following equation [104]:

(2) $$Xc\left(\%\right)=\frac{\Delta H_{f,PCL}}{\Delta H_{PCL}^{0}}\times \frac{1}{w}\times 100$$

where $∆H_{f,PCL}$ is the enthalpy of melting of PCL measured in the DSC tests, $∆H_{PCL}^{0}$ is the heat of melting of 100% PCL (139.5 J g^−1^ [105]), and w is the weight fraction of PCL in the fibers.

Thermogravimetric analyses (TGA) were conducted using a Thermogravimetric Analysis (TGA 2, Mettler toledo AG, Greifensee, Switzerland)) setup under a nitrogen inert atmosphere (50 mL/min). Samples were placed in alumina crucibles and heated from 25 °C to 700 °C at a heating rate of 10 °C/min.

#### 3.5.5. Mechanical Properties

The mechanical properties of the electrospun fibers were determined using an Instron universal testing machine (Model 23-5D, Instron, Norwood, MA, USA) in accordance with the ASTM D638-14 standard [106]. Rectangular specimens measuring 6 cm × 1 cm were cut from the electrospun fiber mats. For each specimen, the thickness and width were measured at three points along its length to calculate average values, for input into testing software. Across all tested specimens, the overall thickness and width were 0.20 (mm) and (10.00 (mm), respectively. Prior to testing, the samples were dried at 37 °C for 24 h and subsequently stored in a partial vacuum desiccator for 3 h. During the tensile test, a total of five specimens (*n* = 5) by formulation were mounted between pneumatic grips equipped with rubber-coated surfaces to prevent mechanical damage. An initial gage length of 40 mm was set, and a slight preload was applied to eliminate slack without elongating the sample. The specimens were elongated at a crosshead speed of 10 mm/min using a 500 N load cell at room temperature until complete mechanical failure occurred. From the resulting stress–strain curves, the Young’s modulus, tensile strength, and strain at break were determined.

#### 3.5.6. Water Absorption and Weight Loss Assays

The water absorption and weight loss behavior of the scaffolds were evaluated in triplicate by immersion in a PBS solution. Square-shaped scaffolds (1.5 cm × 1.5 cm) were cut and placed in 50 mL falcon tubes containing 20 mL of PBS solution (pH 7.3). Then, the tubes were soaked at 37 °C in a shaking incubator. At predetermined time points (1, 7, 14 and 28 days), the scaffolds were removed from the PBS solution and rinsed twice with water. Three independent scaffold samples were analyzed for each formulation at each time point (*n* = 3).

For water absorption measurements, excess surface water was carefully removed, and the wet samples were immediately weighed using an analytical balance. In the case of weight loss studies, the samples were dried under vacuum for 48 h before weighing. The weights of the wet (absorption) and dry (weight loss) samples were used to determine water uptake and weight loss associated with scaffold weight loss over time. The water absorption and weight loss were calculated using Equations (3) and (4), respectively.

(3) $$Water\;absortion\left(\%\right)=\frac{W_{wet\;sample\;t}-W_{dry\;sample\;t}}{W_{dry\;sample\;t}}\times 100$$

(4) $$Weight\;loss\left(\%\right)=\frac{W_{0}-W_{dry\;sample\;t}}{W_{0}}\times 100$$

where $W_{0}$ is the initial weight of the sample before conducting the tests, $W_{wet\;sample\;t}$ corresponds to the weight of the wet sample after a specific immersion time, and $W_{dry\;sample\;t}$ refers to the weight of the dry sample after each drying stage.

### 3.6. In Vitro Cell Viability, Hemolysis, and Antimicrobial Assays

#### 3.6.1. Cell Viability Assay

To evaluate the cytocompatibility of the electrospun scaffolds intended for wound dressing applications, NIH-3T3 murine fibroblasts were selected as a representative skin-related cell model. The cells were obtained from the cell bank of the Immunology and Virology Laboratory, Faculty of Biological Sciences, Universidad Autónoma de Nuevo León (UANL). For maintenance and proliferation, cells were cultured in Dulbecco’s Modified Eagle Medium (DMEM) supplemented with 10% fetal bovine serum (FBS) and 1% antibiotic–antimycotic solution under standard culture conditions at 37 °C in a humidified atmosphere containing 5% CO_2_.

The indirect cytotoxicity assay was performed using extracts of the electrospun materials in accordance with ISO 10993-5 [87], while sample preparation and extraction were conducted following ISO 10993-12 [107]. These standards describe the evaluation of the biological response of cultured mammalian cells exposed to substances released from medical devices or biomaterials.

For this assay, electrospun fibers of PCL, PCL/n-Al_2_O_3_, PCL/starch and PCL/starch/n-Al_2_O_3_ were washed with PBS and sterilized under ultraviolet light for 20 min at 3000 µJ using a CL-1000 Ultraviolet Crosslinker (UVP, Upland, CA, USA). The samples were then washed twice with sterile sodium chloride solution (0.9% *w*/*w*) to remove residual material and exposed again under the same conditions.

For extract preparation, representative portions of each material were incubated in complete DMEM using an extraction ratio of 6 cm^2^/mL. Extraction was performed for 72 h at 37 °C under sterile conditions. After the extraction period, the material samples were removed, and the resulting extracts were collected and used immediately for the cytotoxicity assay without further dilution. Complete DMEM incubated under the same conditions without material was used as the negative control extract.

NIH-3T3 cells were seeded into 96-well plates at a density of 10,000 viable cells per well in a final volume of 100 µL of complete DMEM. After 24 h of incubation to allow cell attachment, the culture medium was removed and replaced with 100 µL of the corresponding material extract. Cells exposed only to the negative control extract were considered the control group. Each material extract was evaluated in triplicate (*n* = 3) after 24, 48, and 72 h of exposure.

The cytotoxicity of each fiber type was evaluated in triplicate at 24, 48, and 72 h. After incubation, at each evaluation time, cell viability was determined using [3-(4,5-dimethylthiazol-2-yl)-5-(3-carboxymethoxyphenyl)-2-(4-sulfophenyl)-2H-tetrazolium] (MTS) assay in combination with phenazine methosulfate (PMS). A working MTS/PMS solution was added at a volume of 20 µL per well, followed by incubation for 4 h at 37 °C in a humidified 5% CO_2_ atmosphere. The absorbance was subsequently measured at 490 nm using a Varioskan™ LUX Multimode Microplate Reader (Thermo Fisher Scientific, Waltham, MA, USA) [108].

Cell viability was calculated relative to the negative control according to the following equation:

(5) $$\mathrm{Cell}\;\mathrm{viability}\;(\%)=\frac{A_{\mathrm{treated}\;\mathrm{cells}}-A_{\mathrm{extract}\;\mathrm{blank}}}{A_{\mathrm{negative}\;\mathrm{control}}-A_{\mathrm{medium}\;\mathrm{blank}}}\times 100$$

Extract blanks containing each material extract and MTS/PMS reagent without cells were included to correct for possible optical interference or direct reduction of the tetrazolium reagent by released components. According to ISO 10993-5, a reduction in cell viability greater than 30% compared with the negative control was considered indicative of a cytotoxic effect; therefore, viability values below 70% were classified as potentially cytotoxic.

#### 3.6.2. Hemocompatibility Assessment by Hemolysis Assay

The hemocompatibility of the electrospun membranes was evaluated using a direct-contact hemolysis assay according to the ASTM F756-17 Standard Practice for Assessment of Hemolytic Properties of Materials [95], with minor modifications based on previously reported methods [109]. Square membrane specimens (1 × 1 cm^2^) were sterilized and individually placed into sterile microcentrifuge tubes containing PBS.

Fresh whole blood was collected from healthy mice into heparinized tubes to prevent coagulation. The blood was diluted with PBS (pH 7.4) according to the ASTM F756 protocol and added to the tubes containing the membrane specimens. The samples were gently mixed to ensure uniform contact between the erythrocytes and the membrane surface and incubated at 37 °C for 3 h under static conditions, with gentle inversion every 30 min to maintain homogeneous cell suspension.

Following incubation, the suspensions were centrifuged at 15,000 rpm for 10 min, and the supernatants were carefully collected. The concentration of free hemoglobin released from lysed erythrocytes was determined by measuring the absorbance of the supernatant at 540 nm using a microplate spectrophotometer.

Blood diluted in PBS (pH 7.4) without contact with any material was used as the negative control (0% hemolysis), whereas blood mixed with deionized water was used as the positive control (100% hemolysis) to induce complete osmotic lysis of erythrocytes. The percentage of hemolysis was calculated according to the following equation:

(6) $$\mathrm{Hemolysis}\;(\%)=\frac{A_{sample}-A_{negative}}{A_{positive}-A_{negative}}\times 100$$

where $A_{sample}$, $A_{negative}$, and $A_{positive}$ correspond to the absorbance values of the test sample, negative control, and positive control, respectively.

According to ASTM F756-17, materials exhibiting <2% hemolysis were classified as non-hemolytic, those producing 2–5% hemolysis as slightly hemolytic, and materials inducing >5% hemolysis as hemolytic. All experiments were performed in triplicate, and the results are expressed as mean ± standard deviation.

#### 3.6.3. In Vitro Cell Migration Assay

The effect of the electrospun scaffold extracts on fibroblast migration was evaluated using an in vitro scratch wound healing assay. Murine fibroblasts (NIH-3T3) were cultured in Dulbecco’s Modified Eagle Medium (DMEM) supplemented with 10% fetal bovine serum and 1% antibiotic-antimycotic solution at 37 °C under a humidified atmosphere containing 5% CO_2_. Cells were seeded in 24-well plates and allowed to reach approximately 90–100% confluence. A linear scratch was generated across the cell monolayer using a sterile 200-µL pipette tip, and detached cells were removed by washing with PBS.

Extracts of the electrospun scaffolds (PCL, PCL/starch, PCL/n-Al_2_O_3_ and PCL/starch/n-Al_2_O_3_) were prepared according to the recommendations of ISO 10993-5, by incubating sterilized samples in complete culture medium for 72 h at 37 °C using the standard extraction ratio. The culture medium was then replaced with the corresponding scaffold extracts. Cells cultured in fresh complete medium served as the positive migration control.

Phase-contrast images were acquired immediately after scratching (0 h) and after 24, 48 and 72 h using an inverted microscope. The wound area was quantified using Image-J software, and the percentage of relative wound closure was calculated with respect to the initial wound area according to:

$$\mathrm{Relative}\;\mathrm{wound}\;\mathrm{closure}\;(\%)=\frac{A_{0}-A_{t}}{A_{0}}\times 100$$

where A_0_ represents the initial wound area and A_t_ the wound area at each evaluation time. Data are presented as mean ± SD from three independent experiments. Statistical analysis was performed using one-way ANOVA followed by Bonferroni’s multiple comparison test, considering *p* < 0.05 as statistically significant.

#### 3.6.4. Protein Expression Analysis

The effect of the electrospun scaffold extracts on proteins involved in fibroblast proliferation and extracellular matrix remodeling was evaluated by a cell-based enzyme-linked immunosorbent assay (ELISA), following the protocol previously reported by Mejía Suaza et al. (2023) with minor modifications consisting only of replacing the primary antibody according to the target protein [110]. Extracts from PCL, PCL/starch, PCL/n-Al_2_O_3_ and PCL/starch/n-Al_2_O_3_ scaffolds were prepared according to ISO 10993-5 by incubating sterilized samples in complete culture medium for 24 h at 37 °C using the recommended extraction ratio.

NIH-3T3 fibroblasts were seeded at 1 × 10^4^ cells per well in 96-well plates and cultured in DMEM supplemented with 10% fetal bovine serum and 1% antibiotic-antimycotic solution. After cell attachment, the culture medium was replaced with the corresponding scaffold extracts, and cells were maintained for 5 days to evaluate the expression of proteins involved in the wound healing process.

At the end of the incubation period, cells were washed twice with PBS and fixed with 5% formaldehyde for 30 min. After washing with PBS, cells were permeabilized using 0.1% Triton X-100 containing 0.1% sodium citrate for 2 min. Non-specific binding sites were blocked with 0.1 M Tris-HCl (pH 7.5) containing 3% bovine serum albumin (BSA) and 10% fetal bovine serum for 1 h.

Cells were incubated overnight at 4 °C with the corresponding primary antibodies against PCNA, TGF-β1, or Collagen type I (1:1000 dilution). After PBS washing, HRP-conjugated goat anti-mouse IgG secondary antibody (1:1000) was added and incubated overnight at 4 °C. Following two PBS washes, TMB substrate was added for 5 min, and the reaction was stopped using 0.16 M sulfuric acid. Absorbance was measured at 450 nm using a Varioskan™ LUX microplate reader (Thermo Fisher Scientific, Waltham, MA, USA).

Protein expression was normalized to β-actin, and results were expressed as relative expression. Data correspond to three independent experiments and were analyzed by one-way ANOVA followed by Bonferroni’s multiple comparison test.

#### 3.6.5. Antibacterial Test Activity

The antibacterial activity was evaluated using an agar disk diffusion assay based on the Kirby–Bauer method [111]. Circular samples or 6 mm in diameter were cut from each electrospun fiber, while a commercial sensi-disk impregnated with gentamicin was used as positive control. All disks were placed on Mueller–Hinton agar plates and were incubated for 24 h at 37 °C with two replicates for each electrospun fiber (*n* = 2). The inhibition zones were measured using a ruler from the center of the disk to the edge of the clear zone. Antibacterial activity was assessed against *Escherichia coli* (Gram-negative) and *Staphylococcus aureus* (Gram-positive) by comparing the inhibition zones.

The percentage of inhibition was calculated according to Equation (7):

(7) $$Inhibition\left(\%\right)=\left(\frac{Inhibition\;zone\;of\;the\;sample(mm)}{Inhibition\;zone\;of\;control(mm)}\right)\times 100\%$$

### 3.7. Statistical Treatment

Quantitative data are presented as the mean ± standard deviation (SD). The sample size (*n*) indicates the number of independent samples or experiments, as specified for each assay and in the corresponding figure legends. The statistical analysis was performed using one-way analysis of variance (ANOVA). The significance of the difference between each group in fiber diameter, water absorption, weight loss, antibacterial activity and mechanical properties were determined using the Bonferroni test. In vitro cell viability was assessed, after the ANOVA analysis, applying Dunnett’s multiple comparison test, and lastly, the data were normalized by the Kolmogorov–Smirnov test. At minimum three fiber samples were used, except for mechanical properties, where five specimens were tested. Statistical significance was defined as * = *p* < 0.05, ** = *p* < 0.01, and *** = *p* < 0.001 were considered significant.

## 4. Conclusions

In this study, we report for the first time the successful fabrication of electrospun PCL/starch/n-Al_2_O_3_ fibers with potential applicability in wound dressing. Polymer scaffolds composed of PCL, PCL/n-Al_2_O_3_, PCL/starch, and PCL/starch/n-Al_2_O_3_ were produced by electrospinning using a side-by-side configuration. SEM analysis confirmed the formation of homogeneous fibrous and porous structures with smooth fibers, with average diameters of 0.37 ± 0.06, 0.61 ± 0.21, 0.86 ± 0.22 and 0.17 ± 0.04 µm for PCL, PCL/n-Al_2_O_3_, PCL/starch and PCL/starch/n-Al_2_O_3_, respectively. These variations in fiber diameter are consistent with the effects of solution properties during electrospinning: starch increased solution viscosity, favoring the formation of thicker fibers, whereas the incorporation of n-Al_2_O_3_ enhanced the electrical conductivity, promoting greater jet stretching and yielding smaller fibers in the ternary system. DSC analysis showed that both n-Al_2_O_3_ and starch inhibit PCL crystallization. Despite this effect, mechanical testing demonstrated that starch and n-Al_2_O_3_ acted as reinforcing phases, significantly increasing Young’s modulus by 157% for PCL/n-Al_2_O_3_ and 321% for PCL/starch compared with neat PCL. Notably, increases in elongation at break were also observed, which may be partially related to morphological features such as fiber diameter and improved stress distribution within the fibrous network. The ternary PCL/starch/n-Al_2_O_3_ scaffold showed the most balanced mechanical behavior, with simultaneous improvements in stiffness and ductility (404% increase in Young’s modulus and 102% increase in elongation at break compared to PCL). Due to the hydrophilic nature of starch and n-Al_2_O_3_, composite scaffolds displayed increased water absorption and accelerated weight loss. The PCL/starch/n-Al_2_O_3_ fibers exhibited the highest weight loss rate, which can be explained by the fact that their lower crystallinity and higher surface area are associated with their smaller fiber diameter.

Cell viability assays using NIH-3T3 fibroblasts cells demonstrated that, although the incorporation of n-Al_2_O_3_ alone increased cytotoxicity, the addition of starch mitigated this effect, whereas PCL/starch/n-Al_2_O_3_ electrospun fibers showed no detectable cytotoxicity. Furthermore, the PCL/starch/n-Al_2_O_3_ scaffold exhibited non-hemolytic behavior, enhanced fibroblast migration, and increased expression of proteins associated with cell proliferation and extracellular matrix remodeling, indicating a favorable biological response. Regarding antibacterial performance, only nanoparticle-containing scaffolds showed activity against both *S. aureus* and *E. coli*, with PCL/starch/n-Al_2_O_3_ exhibiting the highest antibacterial effect.

Overall, these results demonstrate that the rational combination of a hydrophilic biopolymer and inorganic nanoparticles within a side-by-side electrospun architecture overcomes the intrinsic trade-offs of existing systems, enabling the design of multifunctional scaffolds with enhanced mechanical, biological, and antimicrobial properties. This work establishes the development of advanced wound healing materials for PCL/starch/n-Al_2_O_3_ where structural support, degradation behavior, and infection control must be simultaneously achieved. These results justify further studies which explore the therapeutical efficacy onto the wound healing of this nanocomposite using in vivo evaluation in murine preclinical models.

## Figures and Tables

**Table 1:** Crystal size of PCL along the direction (110).

Fibers	Crystal Size (nm)
PCL	34.16
PCL/starch	34.36
PCL/n-Al_2_O_3_	34.39
PCL/starch/n-Al_2_O_3_	32.28

**Table 2:** Crystallization temperature (Tc), melting temperature (Tm), enthalpy of fusion (ΔH_f_) and percent crystallinity of PCL (Xc) for materials based on electrospun fibers.

Scaffold	DSC Results				TGA Results		
	Tc (°C)	Tm (°C)	ΔH_f_ (J/g)	Xc (%)	T_10%_ (°C)	T_max1_ (°C)	T_max2_ (°C)
PCL	29	63	74.5	53	384	–	410
PCL/n-Al_2_O_3_	29	60	69.5	51	348	360	414
PCL/starch	27	60	53.6	51	364	312	410
PCL/starch/n-Al_2_O_3_	27	59	48.9	48	317	314	411

**Table 3:** Fiber composition and composition of the solutions used during electrospinning.

Electrospun Mat	Expected Fiber Composition (PCL/starch/n-Al_2_O_3_) (%)	Solution 1	Solution 2
PCL	100/0/0	FS-A	FS-A
PCL/n-Al_2_O_3_	97/0/3	FS-A	FS-B
PCL/starch	75/25	FS-C	FS-A
PCL/starch/n-Al_2_O_3_	73/24/3	FS-C	FS-B

## Data Availability

Data will be made available on request.
